# Nucleoporins facilitate ORC loading onto chromatin

**DOI:** 10.1016/j.celrep.2022.111590

**Published:** 2022-11-08

**Authors:** Logan Richards, Christopher L. Lord, Mary Lauren Benton, John A. Capra, Jared T. Nordman

**Affiliations:** 1 Department of Biological Sciences, Vanderbilt University, Nashville, TN 37232, USA; 2 Department of Computer Science, Baylor University, Waco, TX 76798, USA; 3 Bakar Computational Health Sciences Institute and Department of Epidemiology and Biostatistics, UCSF, San Francisco, CA 94143, USA; 4 Lead contact

## Abstract

The origin recognition complex (ORC) binds throughout the genome to initiate DNA replication. In metazoans, it is still unclear how ORC is targeted to specific loci to facilitate helicase loading and replication initiation. Here, we perform immunoprecipitations coupled with mass spectrometry for ORC2 in *Drosophila* embryos. Surprisingly, we find that ORC2 associates with multiple subunits of the Nup107-160 subcomplex of the nuclear pore. Bioinformatic analysis reveals that, relative to all modENCODE factors, nucleoporins are among the most enriched factors at ORC2 binding sites. Critically, depletion of the nucleoporin Elys, a member of the Nup107-160 complex, decreases ORC2 loading onto chromatin. Depleting Elys also sensitizes cells to replication fork stalling, which could reflect a defect in establishing dormant replication origins. Our work reveals a connection between ORC, replication initiation, and nucleoporins, suggesting a function for nucleoporins in metazoan replication initiation.

## In brief

Richards et al. show that ORC2, a subunit of the origin recognition complex, interacts with several subunits of the Nup107-160 subcomplex of the nuclear pore. Nucleoporins and ORC2 bind to similar genomic regions, and the chromatin association of ORC2 is dependent on the key nucleoporin Elys.

## Graphical Abstract

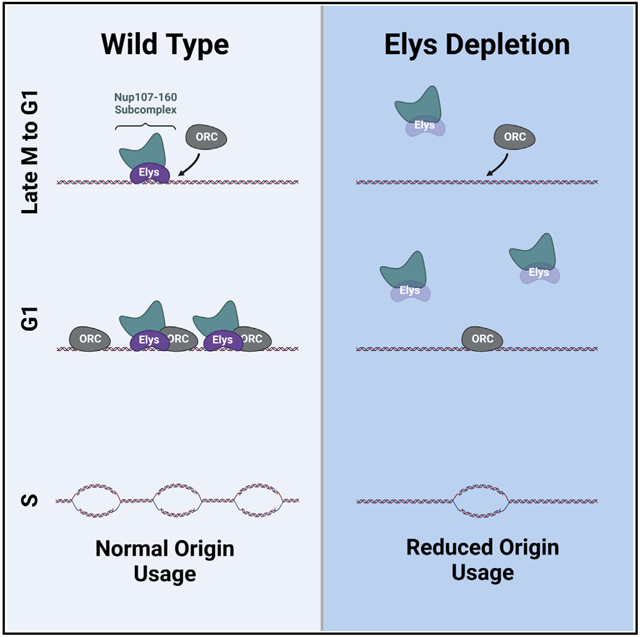

## INTRODUCTION

The origin recognition complex (ORC) binds to thousands of sites throughout the genome to initiate DNA replication (Leonard and Méchali, 2013). The chromatin-bound ORC, together with additional factors, performs the essential function of loading inactive MCM2–7 helicases across the genome in late M and G1 phases of the cell cycle (Fragkos et al., 2015). The distribution of ORC binding sites is critical to define replication start sites and to maintain genome stability, as large genomic regions devoid of replication start sites are prone to breakage upon replication stress (Cha and Kleckner, 2002; Letessier et al., 2011; Miotto et al., 2016; Newman et al., 2013). Additionally, the number and distribution of ORC binding sites and replication start sites can change during development to accommodate cell-type-specific DNA replication programs (Eaton et al., 2011; Hua et al., 2018; Sher et al., 2012). Therefore, studying how ORC is targeted to chromatin is essential to understanding how genome stability is maintained throughout development.

The factors that determine where ORC binds differ across species; however, both DNA sequence and chromatin environment can be important contributors. In *S. cerevisiae*, ORC binding is largely sequence dependent and influenced by nucleosome positioning (Eaton et al., 2010; Wyrick et al., 2001; Xu et al., 2006). While there are a small number of defined initiator sequences in metazoans (Altman and Fanning, 2001; Austin et al., 1999; Lu et al., 2001), ORC binding is largely sequence independent and influenced by both chromatin state and DNA topology (Eaton et al., 2010, 2011; MacAlpine et al., 2010; Miotto et al., 2016; Remus et al., 2004; Vashee et al., 2003). ORC tends to localize to the transcription start sites of active genes (Eaton et al., 2011; MacAlpine et al., 2010). Hallmarks of ORC binding include open regions of chromatin, histone modifications associated with active chromatin, and, in *Drosophila*, sites of cohesion loading (Eaton et al., 2011; MacAlpine et al., 2010; Miotto et al., 2016). Furthermore, in *Drosophila*, specific proteins such as E2f, Rbf, and a Myb-containing protein complex can help recruit ORC to a specific initiation site (Beall et al., 2002; Bosco et al., 2001; Royzman et al., 1999). In humans, ORC-associated protein (ORCA) localizes to heterochromatin and facilitates ORC loading onto chromatin (Shen et al., 2010). The number of ORC binding sites greatly exceeds the number of replication start sites used in a given cell cycle (Cayrou et al., 2011). These excess ORC binding sites license dormant replication origins, which have a critical role in promoting genome stability by ensuring that additional replication start sites are available upon replication stress (Doksani et al., 2009; Ge et al., 2007; Ibarra et al., 2008).

Nucleoporins, or Nups, are typically associated nuclear pore complexes (NPCs) and facilitate the import and export of proteins and macromolecules across the nuclear membrane (for review, see Wente and Rout, 2010). In addition to their canonical function at NPCs, a subset of Nups bind to chromatin and regulate genome structure and function. For example, the Nup Elys binds to chromatin in late mitosis and is required to assemble NPCs onto chromatin prior to their insertion into the nuclear membrane (Franz et al., 2007; Galy et al., 2006; Gillespie et al., 2007; Rasala et al., 2006; Shevelyov, 2020). More recent work, however, has demonstrated that several Nups regulate both transcription and chromatin condensation (Capelson et al., 2010; Kalverda et al., 2010; Kuhn et al., 2019; Panda et al., 2014; Pascual-Garcia et al., 2014, 2017; Raices and D’Angelo, 2017; Vaquerizas et al., 2010). In *Drosophila*, Nup98 binds to distinct regions of the genome, co-localizes with RNA polymerase II, and regulates mRNA levels (Panda et al., 2014; Pascual-Garcia et al., 2014). Furthermore, the genomic localization of Nup98 and Elys correlate with actively transcribed genes (Pascual-Garcia et al., 2017). Tethering the Nups Nup62 or Sec13 is sufficient to decondense chromatin within specific regions of chromatin (Kuhn et al., 2019). Interestingly, this chromatin decondensation correlates with the recruitment of Elys and the PBAP/Brm chromatin-remodeling complex (Kuhn et al., 2019). Many Nups are not permanently anchored to the NPC but, rather, dynamically associate with the NPC throughout the cell cycle (Rabut et al., 2004), and the interaction between Nups and chromatin can occur in the nucleoplasm (Ibarra and Hetzer, 2015). Therefore, it is likely that many Nups have chromatin-related functions independent of the NPC.

In this study, we show that ORC associates with members of the Nup107-160 subcomplex of the nuclear pore. We then show that Nups co-localize with ORC2-binding sites across the genome and that Nups are some of the most enriched chromatin-related factors at ORC sites. We find that depletion of Elys, but not other Nups, reduces the amount of chromatin-bound ORC2 throughout the genome. Importantly, Elys likely promotes ORC2 association independently of its role in promoting chromatin decompaction, as we observe no difference in chromatin accessibility at ORC2 binding sites upon Elys depletion. Finally, we show that depletion of Elys and Nup98-96 sensitizes cells to replication fork inhibition. We propose that Elys is necessary to load the optimal level of ORC on chromatin. Reduction in ORC levels upon Elys depletion could underlie the sensitivity to replication fork stalling by jeopardizing the establishment of dormant origins. Thus, our work provides insight into how the metazoan ORC is recruited to chromatin and defines a replication-associated function of Nups in *Drosophila*.

## RESULTS

### ORC associates with Nups

**Figure 1.**
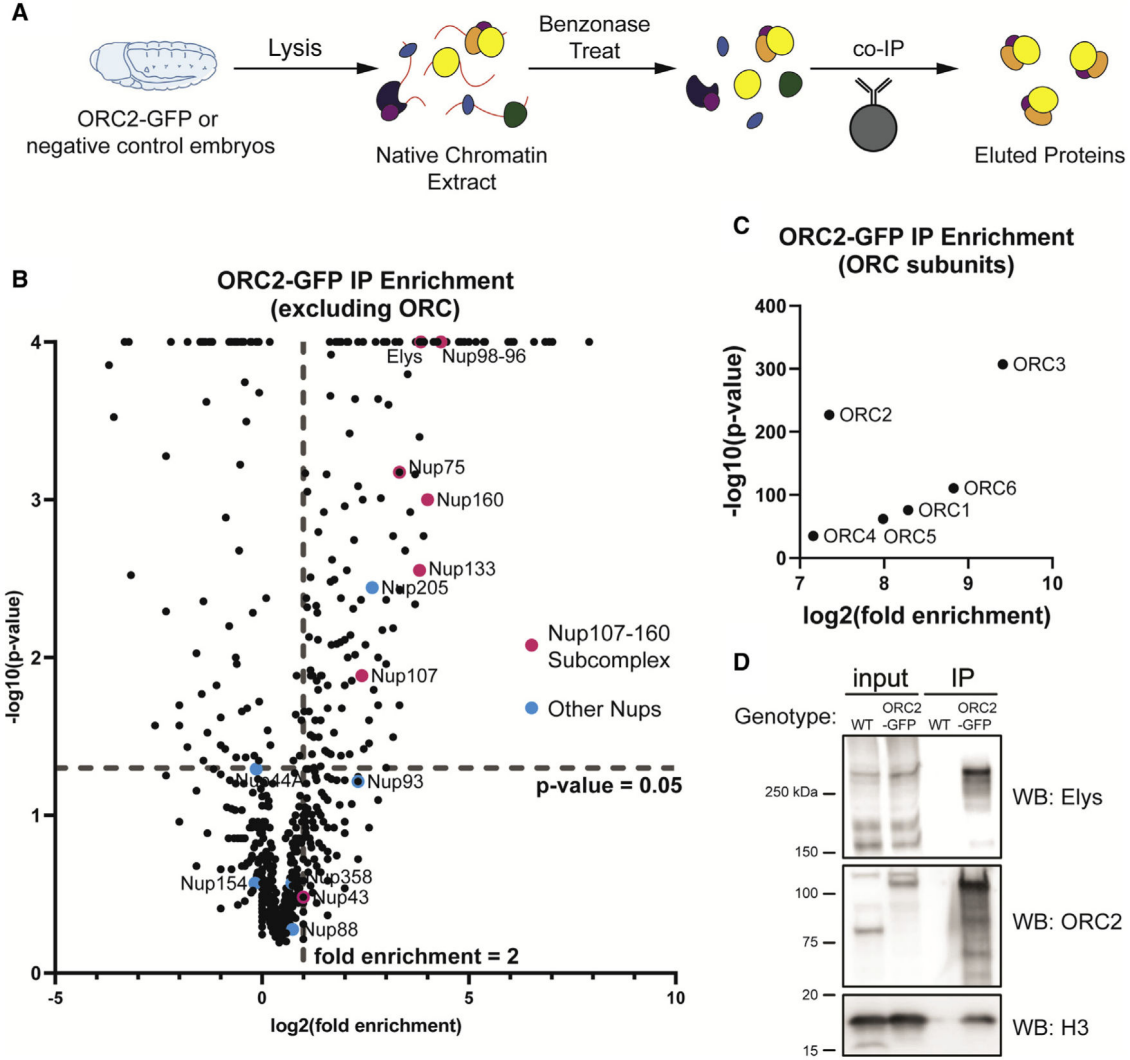
ORC interacts with subunits of the nuclear pore complex (A) Schematic of extract preparation and immunoprecipitation protocol using *ORC2-GFP* or *Oregon R* (negative control) embryos. (B) Average fold enrichment and statistical significance for three biological replicates of GFP-Trap immunoprecipitation (IP) mass spectrometry for *ORC2-GFP*-expressing embryos relative to negative control embryos. Highlighted are all nucleoporin proteins identified by mass spectrometry. Dashed lines indicate significant level cutoffs (<0.05 for p value and ≥2-fold enrichment). (C) Same as (B) but with only ORC subunits. (D) Western blots using anti-ORC2, anti-Elys, or anti-histone H3 antibody on samples derived from the IP.

While a number of chromatin-associated factors are important for ORC genomic binding in metazoans, uncovering factors that facilitate ORC recruitment still remains an under-studied aspect of genome replication (Eaton et al., 2011; Shen et al., 2010). To identify factors that interact with ORC to facilitate ORC binding to chromatin or regulate ORC activity, we immunoprecipitated endogenously tagged ORC2-GFP from *Drosophila* embryo extracts (Figure 1A). Importantly, extracts were benzonase treated to ensure that ORC2-associated proteins were not indirectly bridged by DNA. We used a stringent statistical cutoff to define ORC2-associated proteins (p value of less than 0.05 and a fold enrichment greater than 2; see STAR Methods; Table S1). Using these parameters, we identified all six subunits of ORC (Figure 1C). Surprisingly, we also identified six Nups (Elys, Nup98-96, Nup75, Nup160, Nup133, and Nup107) that were statistically enriched in ORC2-GFP immunoprecipitation (Figures 1B and S1A). Interestingly, five out of the six ORC2-GFP-associated Nups are members of the Nup107-160 complex that form rings on the inner and outer faces of the nuclear pore (Beck and Hurt, 2017). Given that an antibody specific to *Drosophila* Elys was available, we used immunoprecipitation (IP) followed by western blotting to independently validate the association between ORC2 and Elys (Figure 1D).

The two most enriched Nups identified, Elys and Nup98-96, have roles beyond being structural subunits of the nuclear pore (Kuhn et al., 2019; Panda et al., 2014; Pascual-Garcia et al., 2014, 2017). In *Xenopus* extracts, Elys associates with the activated replicative helicase but not ORC (Gillespie et al., 2007). Furthermore, DNA replication is severely inhibited when Elys is depleted from extracts (Gillespie et al., 2007). Given that the *Xenopus* extract system more closely resembles early *Drosophila* embryogenesis, we repeated the ORC2 IPs throughout *Drosophila* embryogenesis to determine if the association between ORC2 and Elys was developmentally regulated. The association between ORC and Elys, however, occurred at multiple time points through embryogenesis and mirrored protein levels (Figure S1B). Taken together, we conclude that ORC2 associates with Elys and several Nups that make up the Nup107-160 subcomplex of the NPC.

### ORC2 binds the same genomic regions as several Nups

**Figure 2.**
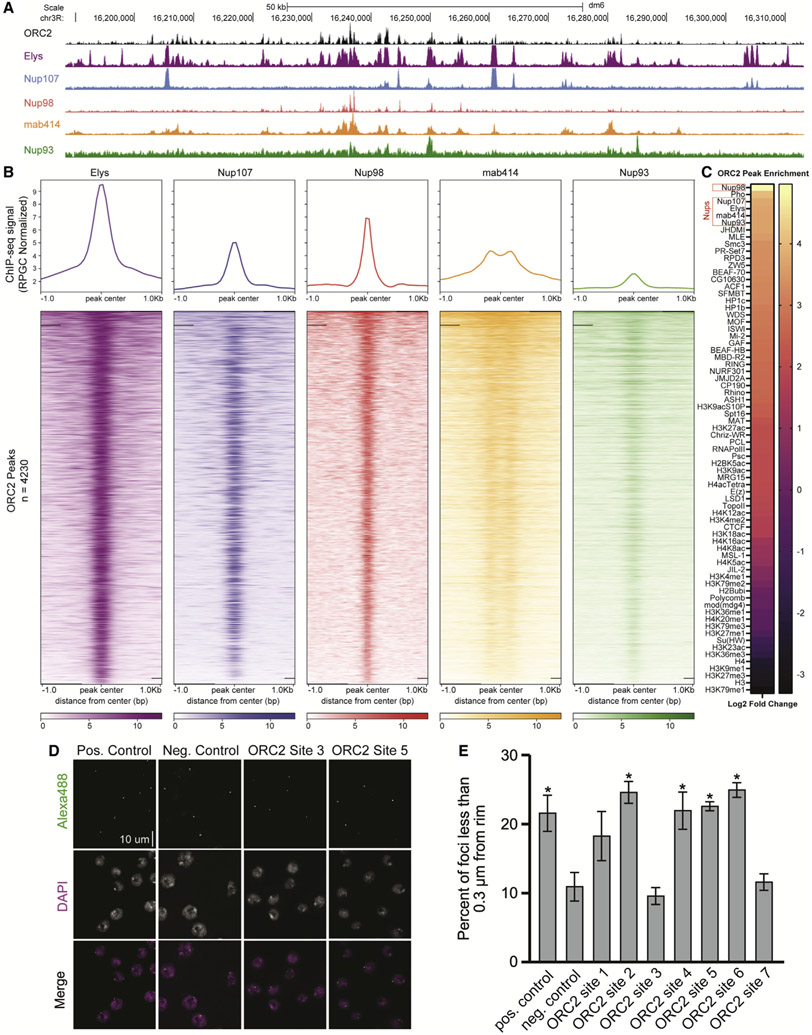
ORC2 binds the same genomic regions as several Nups (A) Representative UCSC genome browser view of ORC2, Elys, Nup107, Nup98, mab414, and Nup93 ChIP-seq (or CUT&RUN) signal generated from previously published data. (B) Enrichment heatmap of ChIP-seq signals sorted by mean occupancy around the center of ORC2 peaks. (C) ORC2 peak enrichment heatmap for chromatin marks, transcription factors, and Nup peaks from previously published data. Log2 fold enrichment for observed overlap relative to expected overlap for each comparison peak set is shown. (D) Representative images of oligopaint performed in S2 cells for one positive (nuclear periphery associating) control site, one negative (nonnuclear periphery associating) control site, and two ORC-binding sites that are also Elys binding sites (Figure S2C for coordinates). (E) Quantification of the percentage of oligopaint foci that were less than 0.3 μm from the nuclear rim for control sites and seven ORC2-Elys binding sites for three biological replicates. Asterisk indicates statistical significance (p < 0.05) relative to the negative control. Error bars denote standard deviation.

Individual Nups bind to distinct chromatin regions to regulate transcription, likely independent of the nuclear pore (Capelson et al., 2010; Kalverda et al., 2010; Kuhn et al., 2019; Panda et al., 2014; Pascual-Garcia et al., 2014, 2017; Raices and D’Angelo, 2017; Vaquerizas et al., 2010). Given that ORC associates with Nups, we asked if ORC and Nups co-localize on chromatin. Using previously published chromatin IP sequencing (ChIP-seq) datasets generated in *Drosophila* S2 cells, we visualized the genomic binding profiles of ORC2 (Eaton et al., 2011) and multiple Nups representing distinct subcomplexes of the nuclear pore (Gozalo et al., 2020; Pascual-Garcia et al., 2017). We also performed CUT&RUN using the mab414 antibody, which recognizes FG repeats found in several Nups, to determine the genomic binding sites of nuclear pores more broadly (Davis and Blobel, 1986). Qualitatively, the binding profile of ORC2 shows extensive overlap with the binding profiles of Elys, Nup107, Nup98, and mab414 (Figure 2A). Next, we quantified ChIP-seq signal of Nups relative to ORC2 peaks and found that Nup and mab414 ChIP-seq or CUT&RUN signal was enriched within ORC2 peaks (Figure 2B). Elys, followed by Nup98, showed the strongest signal across all Nups (Figure 2B). Strikingly, 98% of ORC2 peaks overlap with Elys binding sites (Figure S2A). These data show that ORC2 and Nups bind many of the same genomic regions.

While we observed extensive overlap between ORC2 and Nup binding sites, we wanted to quantitatively measure the significance of this overlap relative to other chromatin-associated factors. To this end, we evaluated the overlap between ORC2 peaks, the available Nup ChIP-seq datasets, and all available S2 cell ChIP-seq datasets available from the modENCODE consortium. For each annotation, we compared the observed overlap with the overlap observed with 1,000 randomly shuffled sets of peaks (Celniker et al., 2009; Contrino et al., 2012). This allowed us to test if the degree of overlap with ORC2 peaks was greater than the expected overlap if peaks were randomly distributed along the genome (STAR Methods). As a proof of principle, our analysis revealed several modENCODE factors that were either enriched or depleted at ORC2 binding sites, consistent with previous work (Eaton et al., 2011). Strikingly, not only were Nups enriched at ORC2 binding sites, they were among the most statistically enriched factors (p value = 0.0001, log2 fold change > 3.5 for Elys, mab414, Nup107, Nup93, and Nup98) out of all 72 datasets we analyzed (Figures 2C and S2B). We also asked if there were any factors enriched at sites that contain Elys and ORC compared with sites that only contain Elys. From this analysis, we found that Polycomb-related factors are enriched at Elys and ORC2 binding sites relative to Elys-only binding sites (Figure S2E). Taken together, we conclude that Nup binding sites show significant overlap with ORC2 binding sites genome wide.

Nups also bind chromatin when in complex with nuclear pores (Kadota et al., 2020; Kuhn and Capelson, 2019). Given this, we were curious if the ORC2 binding sites that overlap with Nup binding sites required localization to the nuclear pore, suggesting that the interaction between ORC and Nups occurs at NPCs. To formally test this, we selected seven 10 kb regions that were positive for both Elys and ORC2 binding (ORC2 sites 1–7) and generated oligopaint probes specific to these sites (Figures S2C and S2D). We then measured the proximity of these seven sites to the nuclear periphery. If ORC binding and co-localization with Nups requires a functional nuclear pore, we would expect these sites to be enriched at the nuclear periphery. This, however, is not the case. Just over half of the sites we tested were found in close proximity to the nuclear rim (Figures 2D and 2E). Given that ORC2/Elys binding sites are not required to be at the nuclear periphery, this suggests that the ORC/Nup association occurs independently of the nuclear pore.

### ORC binding to chromatin partially depends on Elys

So far, we have shown that ORC physically associates with the members of the Nup107-160 subcomplex and that there is a high degree of co-localization between ORC and Nups on chromatin. To determine if there is a functional relationship between ORC and Nups, we asked if the chromatin association of ORC is dependent on Nups. To this end, we depleted either GFP (negative control), ORC2, Elys, or Nup98-96 using RNA interference (RNAi) in *Drosophila* S2 cells. Nup98-96 was selected as a control as these genes are transcribed into a single mRNA, which is translated into a larger precursor protein that is ultimately cleaved to produce Nup98 and Nup96 (Fontoura et al., 1999). Therefore, RNAi against Nup98-96 reduces the steady-state protein level of both Nup98 and Nup96 (Fontoura et al., 1999). Depletions were verified by western blotting against Elys and ORC2 (Figures S3A and S3B).

**Figure 3.**
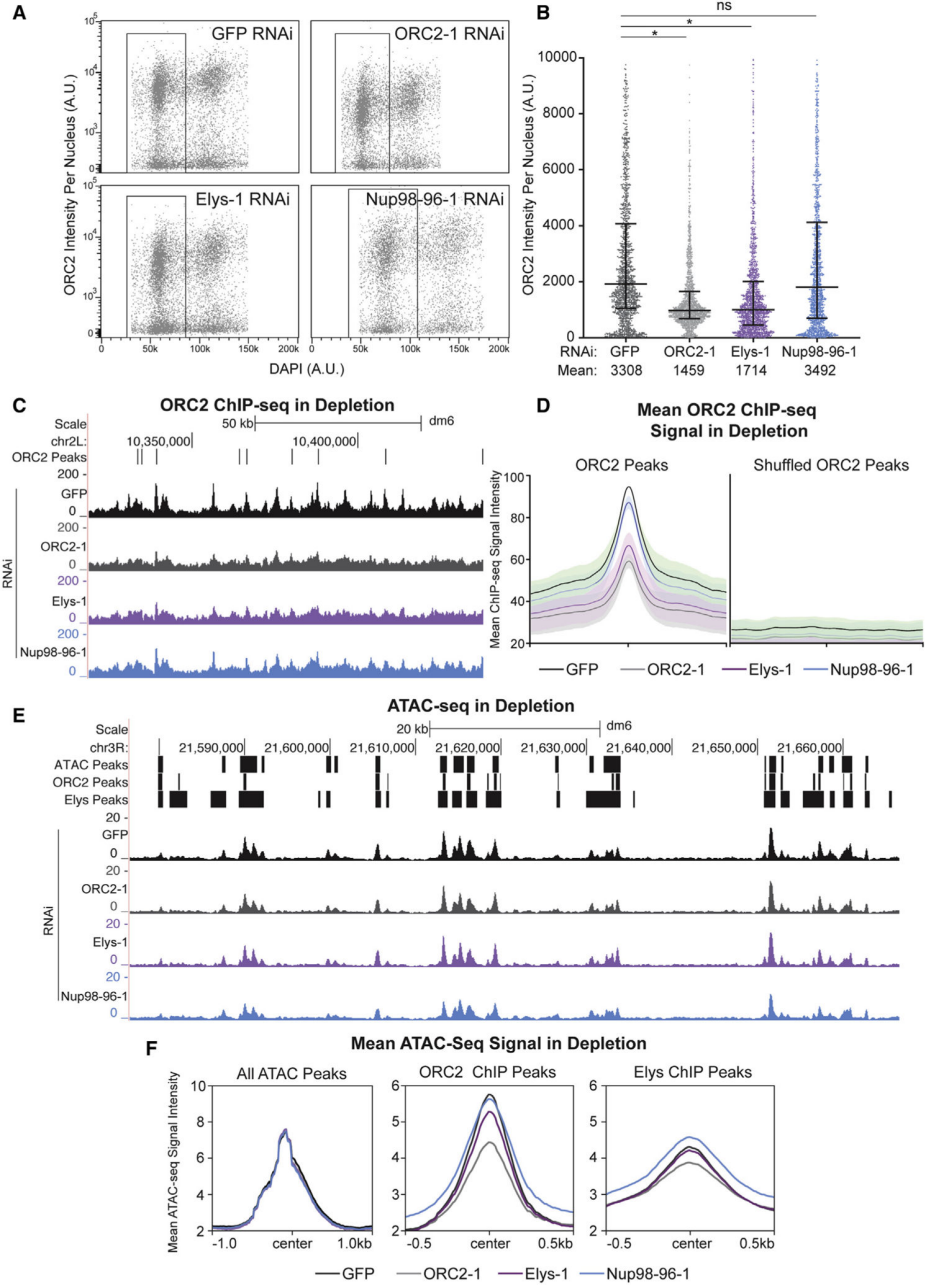
ORC’s chromatin association depends on Elys (A) Horseshoe plot of nuclei with DNA content (DAPI) plotted against ORC2 intensity for each depletion from one replicate. Black box indicates G1 population of nuclei used for the quantification in (B). A.U., arbitrary units. (B) Quantification of ORC2 intensity in 1,500 randomly selected G1 nuclei from three biological replicates. Asterisk indicates p < 0.0001 relative to the negative control. NS, no significance. (C) Representative UCSC genome browser view of ORC2 ChIP-seq profiles for each depletion. ORC2 binding sites (ORC2 peaks, defined by Eaton et al., 2011) are indicated by black bars. (D) Quantification of mean ORC2 ChIP-seq signal within defined ORC2 peaks or shuffled ORC2 peaks, centered on ORC2 peaks or shuffled ORC2 peaks, respectively, for two biological replicates. (E) Representative UCSC genome browser view of ATAC-seq for each depletion for one biological replicate. ATAC-seq peaks, ORC2 ChIP-seq peaks, and Elys ChIP-seq peaks are indicated by black bars. (F) Quantification of mean ATAC-seq signal for either all ATAC-seq peaks (n = 12,771), ORC2 ChIP-seq peaks (n = 4,280), or Elys ChIP-seq peaks (n = 12,048) centered on their respective peaks. Note that the scales are different for all ATAC-seq peak plots.

Given that Elys binds to chromatin to promote the decondensation of chromatin (Kuhn et al., 2019), we hypothesized that Elys, and perhaps other Nups, could promote ORC binding to chromatin, as ORC preferentially associates with open and active regions of chromatin (Eaton et al., 2011; MacAlpine et al., 2010). To test this, we quantified the amount of chromatin-bound ORC2 in G1 phase nuclei in GFP, ORC2, Elys, or Nup98-96 depletions using quantitative flow cytometry (Matson et al., 2017) (see Figure S3C for gating example; Figures 3A and 3B). G1 phase nuclei were selected because any changes in ORC2 chromatin association should be most apparent in this stage, as ORC is loaded in late M and G1. In ORC2-depleted control nuclei, we observed significantly less ORC2 on chromatin, as expected. Consistent with our hypothesis, we observed significantly less chromatin-associated ORC2 in G1 phase upon Elys depletion relative to control cells (Figures 3A and 3B). Cell-cycle analysis revealed that the reduction in ORC2 loading was specific to G1 (Figure S3F). To ensure different cell populations were not skewing the data, we quantified only ORC2-positive nuclei and still observed reduced ORC2 chromatin association (Figure S3H). Interestingly, there was no reduction in chromatin-associated ORC2 upon Nup98-96 depletion, suggesting that not all Nups contribute to ORC loading onto chromatin (Figures 3A and 3B). In fact, depleting Nup107 and Nup160 (both members of the Nup107-160 subcomplex) did not affect ORC2 chromatin association, indicating that the reduction of chromatin-bound ORC2 is not a generic defect of depleting Nups (Figures 3A, 3B, and S3E). As an important control, we performed the same experiment with a second set of double-stranded RNAs (dsRNAs) against ORC2, Elys, and Nup98-96 to eliminate the possibility that our observations are due to nonspecific effects from the dsRNA (Figure S3D). Additionally, we determined that induction of the RNAi machinery itself does not reduce ORC2 chromatin association (Figure S3G). Finally, there was no reduction in histone H2B signal across the depletions, indicating that the reduction we observe is specific to ORC2 (Figures S3I and S3J). Taken together, we conclude that proper ORC2 chromatin association is dependent on Elys and that the reduction in ORC2 chromatin association is not a general defect caused by Nup depletion.

Next, we asked if the reduction of chromatin-bound ORC2 upon Elys depletion occurs throughout the genome or if only specific genomic regions or ORC2 binding sites are affected. To answer this, we performed ChIP-seq using an ORC2 antibody in *Drosophila* S2 cells that were depleted for either GFP, ORC2, Elys, or Nup98-96. We then quantified the ChIP-seq signal intensity within previously identified ORC2 binding sites throughout the genome (Eaton et al., 2011). For our positive control, we observed less ORC2 ChIP-seq signal in the ORC2 depletion relative to the GFP negative control (Figures 3C and 3D). Consistent with our flow cytometry results, there was less ORC2 ChIP-seq signal in the Elys depletion but not in the Nup98-96 depletion (Figures 3C and 3D). Furthermore, we observed a reduction in ORC2 signal throughout the genome, indicating that depletion of Elys impacts all ORC2 binding sites. To ensure that the reduction in ORC2 ChIP-seq signal was specifically within ORC2 peaks and not a general trend throughout the genome, we shuffled all ORC2 peaks across the genome and found no difference in the mean ORC2 ChIP-seq signal (Figure 3D). Therefore, the reduction of signal is specific to ORC2 binding sites (Figure 3D). Taken together, we conclude that depleting Elys results in less ORC2 binding throughout the genome and that Elys, but not the other Nups tested, facilitates ORC2 loading onto chromatin.

Given that Elys is known to promote chromatin decondensation, one possibility is that Elys facilitates ORC loading indirectly by promoting chromatin accessibility, as ORC binds to open chromatin (Eaton et al., 2011; MacAlpine et al., 2010). To test this possibility, we performed assay for transposase-accessible chromatin with high-throughput sequencing (ATAC-seq) in RNAi-treated cells to measure chromatin accessibility within each depletion (Figure 3E and S3K). Importantly, there was no global change in accessibility when comparing all ATAC-seq peaks throughout the genome upon Elys, ORC, or Nup98-96 depletions (Figures 3F and S3L). When comparing accessibility specifically within ORC2 binding sites, we noticed a significant reduction in accessibility upon ORC2 depletion (Figures 3F and S3L). ORC can directly promote chromatin accessibility at ORC binding sites (Eaton et al., 2010), which could drive the reduced chromatin accessibility at ORC2 binding sites. Interestingly, depleting Elys or Nup98-96 is not sufficient to cause a significant reduction in chromatin accessibility at ORC2 binding sites (Figures 3F and S3K-S3L). Together, these data argue that the reduction in ORC chromatin association upon Elys depletion may not be driven by changes in chromatin accessibility.

### Nup depletion sensitizes cells to fork stalling

Given that ORC associates with Nups, co-localizes with Nups on chromatin, and that the association of ORC with chromatin is partially dependent on Elys, we wanted to ask if depletion of Elys and Nup98-96 affects cell-cycle progression and/or genome stability. We reasoned that if ORC loading on chromatin was compromised, then we may observe a defect in S phase entry. Therefore, we pulsed cells with EdU and measured the fraction of cells in G1, S, and G2/M based on their DNA content and EdU status by flow cytometry. In our ORC2 depletion, which serves as a positive control, we saw a modest increase in G1 cells and reduction in S phase cells relative to the GFP negative control, consistent with a defect in S phase entry (Figures S4A and S4B). The modest effect is expected since excess ORC is loaded onto chromatin to ensure sufficient replication start sites to complete DNA replication (Kawabataet al., 2011). Depletion of Elys, however, did not significantly alter the cell-cycle profile relative to the negative control (Figures S4A and S4B). Given the modest effect observed with the ORC2 depletion, and the level of ORC still associated with chromatin upon Elys depletion (Figures 3A-3D), this was not entirely unexpected. Depletion of Nup98-96, however, drastically reduced the fraction of cells in S phase while increasing the fraction of cells in G1 and G2/M (Figures S4A and S4B). Depletion of Nup98-96 did not significantly affect the level of chromatin-bound ORC (Figures 3A-3D). Therefore, we conclude that Nup98-96 influences cell-cycle progression independently of ORC chromatin association. Given that Elys functions at kinetochores during mitosis in mammalian cells and meiosis in *C. elegans* (Galy et al., 2006; Rasala et al., 2006), we measured the impact depletion that Elys, ORC2, or Nup98-96 has on mitotic index using immunofluorescence with an anti-phospho (Ser10) histone H3 antibody (PH3). We found that depletion of Elys had no effect on mitotic index, suggesting that Elys may not have the same mitotic roles in *Drosophila* (Figures S4D and S4E).

**Figure 4.**
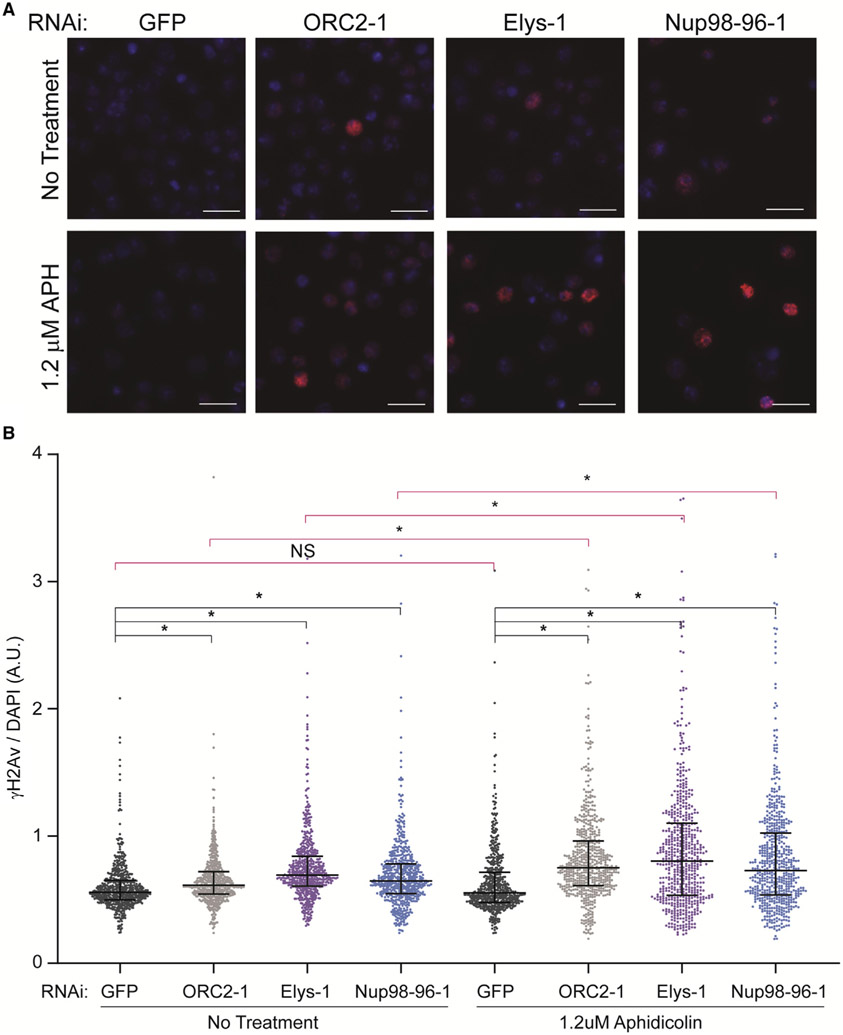
Nup depletion sensitizes cells to fork stalling (A) Representative images of ɣH2Av immunofluorescence performed on RNAi-treated cells with or without aphidicolin treatment. Blue: DAPI. Red: ɣH2Av. Scale bar: 10 uM. (B) Quantification of (A). ɣH2Av and DAPI intensity for 600 total cells randomly selected from two biological replicates were quantified for each depletion with and without aphidicolin treatment. Black bars compare each depletion with negative control (GFP). Pink bars compare untreated cells to aphidicolin-treated cells (GFP untreated versus GFP treated, for example). Asterisk denotes p < 0.0001. NS, no significance. Median and interquartile range are shown.

Excess replication start sites are not always essential during an unperturbed S phase but become critical upon replication stress (Alver et al., 2014). This is largely due to the need to fire dormant replication origins to complete DNA synthesis when replication is perturbed (Doksani et al., 2009; Ge et al., 2007; Ibarra et al., 2008). Given that we observed a reduction of chromatin-bound ORC but no change in the percentage of cells in S phase in an Elys depletion, we hypothesized that a reduction in chromatin-bound ORC could lead to a defect in dormant origin firing. While we attempted to perform DNA combing to measure inter-origin distance, we were unable to measure IdU incorporation in *Drosophila* S2 cells and, therefore, could only measure single CldU tracks, which is not ideal for measuring inter-origin distance (data not shown; Munden et al., 2022). If there are insufficient dormant origins upon an Elys depletion, then Elys-depleted cells should be sensitive to replication fork inhibition as there are less origins available to rescue stalled replication forks (Alver et al., 2014; Doksani et al., 2009; Ge et al., 2007; Ibarra et al., 2008). Therefore, we treated cells depleted for GFP, ORC2, Elys, or Nup98-96 with a low dose of aphidicolin and measured the level of ɣH2Av (the *Drosophila* equivalent of ɣH2Ax in mammals) by quantitative immunofluorescence. We chose a dose of aphidicolin that did not increase the level of DNA damage, as measured by ɣH2Av, in our negative control (GFP) but did cause a modest increase in the fraction of cells in S phase (Figures 4A, 4B, and S4C). We found that depletion of Elys and Nup98-96 alone caused a modest increase in DNA damage (Figures 4A and 4B). Upon aphidicolin treatment, however, there was a significant increase in the amount of DNA damage both relative to the negative control (Figures 4A, bottom panel, and 4B, right) and relative to the untreated depletions (Figures 4A and 4B, pink bars). From these findings, we conclude that the sensitivity to aphidicolin we observe is consistent with the possibility that dormant origin firing is reduced in an Elys depletion.

## DISCUSSION

Our results show that ORC interacts with members of the Nup107-160 subcomplex of the nuclear pore, most notably the Nups Elys and Nup98-96, establishing a link between replication initiation and Nups. Elys, Nup98, Nup93, Nup107, and FG-repeat-containing Nups are enriched at ORC2 binding sites, and Nups are among the most significantly enriched chromatin factors at ORC2 binding sites. Strikingly, 98% of ORC sites are also Elys binding sites. Not all these sites are localized to the nuclear periphery, suggesting that the associations between ORC and Nups are likely occurring off pore. Furthermore, if ORC and NPCs were present in the same protein complex, we would have expected to identify Nup subunits more broadly rather than just a subset of Nups. Therefore, our observations are most consistent with a model where Elys, and possibly other members of the Nup107-160 subcomplex, associate with ORC independently of the nuclear pore. This would be consistent with previously published data where Elys and other Nups perform chromatin-related functions beyond their canonical role in the NPC.

Based on our present findings, we argue that Elys functions to load ORC onto chromatin. Importantly, depletion of other Nups, including members of the Nup107-160 subcomplex, do not reduce the amount of ORC on chromatin. This reveals two important points. First, the reduction in chromatin-associated ORC upon Elys depletion is not a generic effect of altered NPC function. Second, out of the Nups tested, the ability to promote ORC loading seems to be specific to Elys. We do not rule out the possibility, however, that other Nups could contribute to ORC loading either independently or together with Elys. Interestingly, Elys and ORC both bind to chromatin in late M phase. It is possible that Elys, or another Nup, directly or indirectly interacts with ORC late in mitosis to facilitate ORC binding on chromatin by providing a molecular bridge between chromatin remodeling and ORC. While we did not observe a global change in chromatin accessibility upon Elys depletion, it is possible that Elys, together with its known interactor PBAP, could generate a nucleosome-free region that would be optimal for ORC binding. If this happens specifically in late M phase, then it would be difficult to measure changes in chromatin accessibility by ATAC-seq from an asynchronous population of cells.

Given that the number and distribution of loaded helicases is necessary to maintain genome stability, depletion of Elys could compromise genome integrity due to a defect in origin licensing. Consistent with this, depletion of Elys shows an increased sensitivity to replication fork stalling. One possible explanation is that, upon Elys depletion, there is insufficient ORC to promote dormant origin licensing. Alternatively, depletion of Nups could result in fork stalling through mechanisms independent of replication initiation (Kosar et al., 2021). *ORC2* mutants in *Drosophila* have cell-cycle-related phenotypes and altered replication timing (Loupart et al., 2000). Given that nuclear organization is coupled to replication timing (Smith and Aladjem, 2014), depleting Elys may cause changes in replication timing that indirectly influence fork stalling. Interestingly, we observe a similar sensitivity to fork stalling in the Nup98-96 depletion. We predict that this occurs through a different mechanism than the Elys depletion, however, as Nup98-86 depletion results in a stark reduction in cells in S phase but does not significantly change ORC levels. Nup98-96 depletion could affect helicase activation, explaining the Nup98-96-depletion phenotypes. Additionally, failure to fire dormant replication origins would explain the increased sensitivity to fork stalling. Understanding how Nups differentially affect genome duplication and stability is an exciting area of future research.

### Limitations of study

While we demonstrated that ORC and Nups associate and bind the same genomic regions and that depleting Elys is sufficient to reduce the chromatin association of ORC2, there are several unanswered questions. First, it is not clear what the exact direct interactions are between Nups and ORC. While our data suggest that Elys would be a good candidate for subsequent interaction studies, it is unknown if Elys, or any other Nup, directly interacts with ORC or if the interaction is bridged by additional factors. Even if there is a direct interaction between Elys and ORC, it could be regulated by post-translational modifications during the cell cycle. Also, it is still unclear when Nups and ORC associate during the cell cycle. Given that both Nups and ORC associated with chromatin starting late in mitosis, this interaction could be confined to a short window within the cell cycle. Our data show that depletion of Elys causes a reduction in ORC binding, which could lead to an increase in inter-origin distance. Due to technical limitations, however, we are unable to measure this directly. Lastly, the mechanism that Elys or other Nups use to promote ORC binding across the genome still remains to be determined. Understanding the molecular interactions between Nups and ORC will be critical to understanding how ORC is recruited to chromatin to ensure faithful DNA replication.

## STAR★METHODS

### RESOURCE AVAILABILITY

#### Lead contact

Further information and requests for resources and reagents should be directed to and will be fulfilled by the lead contact, Jared Nordman (jared.nordman@vanderbilt.edu).

#### Materials availability

Antibodies and plasmids generated in this study will be provided upon request without restriction.

#### Data and code availability

- All sequencing data have been deposited at GEO and are publicly available as of date of publication. All mass spectrometry raw and processed data have been deposited on ProteomeXChange. Accession numbers are listed in the key resources table. Original western blot images have been deposited at Mendeley and are publicly available as of date of publication. The DOI is listed in the key resources table. Microscopy and flow cytometry data reported in this paper will be shared by the lead contact upon request.
- This paper does not use any original code.
- Any additional information required to reanalyze the data reported in this paper is available from the lead contact upon request.

### EXPERIMENTAL MODEL AND SUBJECT DETAILS

#### Drosophila cells

*Drosophila melanogaster* S2 cells were provided by Drosophila Genomics Resource Center (DGRC stock number 181). Cells are wildtype and derived from embryonic tissue. Cells were maintained following DGRC guidelines. Cells were grown at 25°C and in Schneider’s medium (Gibco 21720024) supplemented with 10% heat-inactivated fetal bovine serum (ThermoFisher A3840001) and penicillin-streptomycin (ThermoFisher 15140122). Cells were passaged every 3–5 days and maintained at a concentration of 3 × 10^6^-1x10^7^ cells/mL.

#### Fly lines

*ORR* flies were a gift from Terry Orr-Weaver (Whitehead Institute). *ORC2-GFP* flies were a gift from Shelby Blythe. CRISPR was used to integrate *GFP* at the endogenous ORC2 gene locus. Flies were maintained in population cages at 25°C and fed wet yeast on grape agar plates daily.

### METHOD DETAILS

#### Immunoprecipitations

Immunoprecipitations were performed on three biological replicates of both *ORC2-GFP* and *ORR* embryos. For each replicate, 0.5 g of embryos aged 18–24 h were collected, dechorionated, and flash frozen. Frozen embryos were ground thoroughly with a mortar and pestle in liquid N2. Ground embryos were then resuspended in NP40 Lysis Buffer (50 mM Tris HCl pH 8.0, 150 mM NaCl, 1% NP40, 1 mM EDTA, 1 mM EGTA, with 2X cOmplete^™^ Protease Inhibitor Cocktail EDTA-free (Millipore Sigma)). The embryonic extract was treated with benzonase nuclease (Millipore #7066) at a final concentration of 30 U/mL for 30 min at 4°C. After benzonase treatment, the extract was centrifuged at 4,000 rcf for 5 min. The supernatant was then used for the immunoprecipitation.

Prior to the immunoprecipitation, GFP Trap magnetic agarose beads (Chromotek #gtma-10) were washed and equilibrated with NP40 lysis buffer. Beads were added to extract and incubated for 2 h at 4°C. After the 2 h, beads were isolated and washed with 4 times with NP40 lysis buffer. Beads were then resuspended in 2× Laemmli sample buffer (Biorad #1610737) and boiled at 95°C for 20 min to elute protein.

#### Mass spectrometry

Eluates in Laemmli buffer were methanol/chloroform precipitated. After precipitation, immunoprecipitated samples were separated on a 4-12% NuPAGE Bis-Tris gel (Invitrogen), proteins were resolubolized in 5% SDS and prepared using S-trap (Protifi) using manufacturer’s protocol. Resulting peptides were desalted via C18 solid phase extraction and autosampled onto a 200 mm by 0.1 mm (Jupiter 3 micron, 300A), self-packed analytical column coupled directly to an Q-exactive+ mass spectrometer (ThermoFisher) using a nanoelectrospray source and resolved using an aqueous to organic gradient. Both the intact masses (MS) and fragmentation patters (MS/MS) of the peptides were collected in a data dependent manner utilizing dynamic exclusion to maximize depth of proteome coverage. Resulting peptide MS/MS spectral data were searched against the Drosophila protein database using SEQUEST (Yates et al., 1995). Identifications were filtered and collated at the protein level using Scaffold (Proteome Software).

Search results and peptide counts were refined in Scaffold using the following parameters: protein threshold false discovery rate = 5% minimum number of peptides ≥2, and a peptide threshold false discovery rate = 5%. Scaffold was used to perform a Fisher’s Test for each individual protein identified, comparing ORC2-GFP to the ORR negative control for all three replicates. For visualization purposes, p values ≤ 0.0010 were rounded to 0.0010 in Figure 2B. For Figure 2C, p values for ORC subunits were generated by a Fisher’s Test using R. Fold enrichment was calculated using the raw spectrum counts for individual proteins over the negative control. Volcano plots visualizing p values and fold enrichment were made using GraphPad Prism.

#### Western blotting

The presence of ORC2 in the elute was confirmed prior to conducting mass spectrometry by SDS-PAGE followed by a Western blot for ORC2 using anti-ORC2 antibody. Briefly, samples were boiled and loaded onto a 4–15% Mini-PROTEAN TGX Stain-Free Gel (Biorad). After electrophoresis, the gel was activated and imaged using a BioRad ChemiDoc^™^ MP Imaging System following manufacturer recommendations. Protein was transferred to a low fluorescence PVDF membrane using a Trans-Blot Turbo Transfer System (BioRad). Membranes were blocked with 5% milk in TBST (140 mM NaCl, 2.5 mM KCl, 50 mM Tris HCl pH 7.4, 0.1% Tween 20). Blots were incubated with either anti-ORC2 antibody at 1:1000, anti-Elys antibody at 1:250, or HRP anti-Histone H3 antibody (abcam #ab21054) at 1:1000 overnight at 4°C. After primary antibody incubation, blots were washed, incubated secondary HRP antibody (Jackson Labs 711-035-150), washed once more and imaged. For the quantification shown in Figure S4B, signal for either Elys or ORC2 was normalized to H3 for each depletion for three biological replicates.

#### Antibody generation

Full length ORC2 tagged with Maltose Binding Protein (MBP) was expressed and purified from *E. coli*. Briefly, ORC2 was cloned into the pLM302 expression vector. The expression construct was transformed into Rosetta 2 (DE3) cells (Novagen) and cultures were induced with IPTG and His-MBP-ORC2 was purified on Ni-NTA beads (BioRad). Purified protein was injected into rabbits for serum generation and collection (Cocalico Biologicals). For affinity purification, serum was first passed over an MBP column to deplete MBP-specific antibodies and the flow through fraction was passed over a column of MBP-ORC2 and eluted. An Elys-specific antibody was generated as previously described (Pascual-Garcia et al., 2017) The C-terminal fragment of Elys, amino acids 1766-2110, was cloned into pLM302 vector, expressed in *E. coli* Rosetta cells and purified using the same techniques described above.

#### Oligopaint fluorescent in situ hybridization (FISH)

Oligo pools were generated using the PaintSHOP application (Hershberg et al., 2021) from the Drosophila dm6 reference genome. A complete list of oligos can be found in Data S1, Table S2. Oligopaint probe production and FISH was performed largely as previously described (Nguyen and Joyce, 2019). Oligo pools were resuspended 50 mL of ddH_2_0 and 1 mL was used for an initial PCR amplification along with 2.5 μL of 10 μM forward (GCGTTAGGGTGCTTACGTC) and reverse (CACCTCCGTCTCTCACCT) primers, 25 μL 2X Q5 master mix, and 19 μL ddH_2_0 with 30 s 98°C denaturation, 30 s 55°C annealing, and 30 s 72°C extension steps repeated 34 times. The PCR product was purified using a MagExtractor PCR & Gel clean up kit (Toyobo NPK-601) according to the manufacturer’s protocol and resuspended in 20 μL ddH_2_0. A secondary amplification was performed by mixing 1 μL of the first PCR product with 100 μL 2X Q5 master mix, 10 μL of 10 μM forward B and reverse B primers, and 79 μL ddH_2_0. The PCR was performed using the same program as described above, and subsequently purified as described and resuspended in 30 μL ddH_2_0. A Megascript T7 (ThermoFisher AM1334) reaction was performed by mixing 14 μL of the secondary PCR product with 4 μL of ATP, CTP, GTP, and UTP solutions and 4 μL reaction buffer, 2 μL RNase inhibitor, and 4 μL RT mix. The T7 reaction was incubated overnight at 37°C. A Maxima H Minus RT (ThermoFisher EP0752) reaction was setup by mixing 40 μL of the T7 reaction with 30 μL 100 uM forward B primer, 19.2 μL 100 mM dNTPs, 60 μL 5X RT buffer, 3 μL RNase inhibitor, 4 μL Maxima H Minus RT, and 143.8 μL ddH_2_0; this was incubated at 50°C for 3.5 h. RNA was degraded by adding 150 μL 0.5 M EDTA and 150 μL NaOH to the reaction, then heating at 95°C for 5 min. The DNA was cleaned and concentrated using a DNA Clean & Concentrator-100 kit (Zymo Research, D4029) and the DNA was resuspended in 90 μL ddH_2_0. The concentration ranged from 200 to 400 ng/μL. Pools were stored at −20°C until use.

For FISH experiments, S2 cells were concentrated and incubated in 100 μL Schnieder’s Drosophila media +10% FBS on polylysine-coated slides for 1–2 h in a humid chamber underneath a strip of parafilm the size of a cover slip. The media was then aspirated and the slides were incubated in freshly-prepared fixative solution (1X PBS and 4% paraformaldehyde) after transferring to coplin jars for 10 min at RT. Slides were washed in 1X PBS then incubated in freshly-prepared 0.5% Triton X-100 for 15 min at RT. Slides were rinsed with 1X PBS then dehydrated with successive incubations with 70%, 90%, and 100% ethanol for 2 min each at RT. Slides were then washed with 2X SSCT (2X SSC and 0.1% Tween 20) for 5 min. Next the slides were incubated with 2X SSCTF (2X SSC, 0.1% Tween 20, and 50% formamide) pre-heated to 90°C for 3 min in a new coplin jar that was also pre-heated. Slides were next incubated in 2X SSCTF at 60°C for 20 min (also pre-warmed). During this incubation, hybridization mix was prepared by vigorously mixing 300 μL formamide, 120 μL 50% dextran sulfate and 60 μL 20X SSC. 20 μL of this hybridization mix was then mixed with 4.5 μL oligo pool (at 200–300 ng/μL) along with 0.5 μL 100 mM dNTPs, which was a sufficient quantity for one slide. Slides were dried for 5 min, then the hybridization mix + probe was added on top of the fixed cells. This was covered with a cover slip and sealed with rubber cement and dried for at least 20 min at RT. Slides were placed into a humid slide incubator and heated to 92°C for 3 min, then incubated overnight at 37°C. The next day cover slips were carefully removed and the slides were washed with 2X SSCT (pre-warmed) at 60°C for 15 min, then 2X SSCT for 10 min at RT, then 0.2X SSCT for 10 min at RT. New hybridization mix was prepared as before, and 120 μL was mixed with 29.75 μL ddH_2_0 and 0.25 μL 100 mM secondary fluorescent oligo probe (sequence of AAGCACCCTAACGCTTCACGATCCAT covalently linked to Alexa Fluor 488 dye), which was sufficient for 5 slides. 25 μL of this mix was then added on top of the fixed cells and sealed with a cover slip and rubber cement and the slides were incubated at RT for 1–2 h. Cover slips were carefully removed and the slides were washed with 2X SSCT (pre-warmed) at 60°C for 15 min, then 2X SSCT for 10 min at RT, then 0.2X SSCT for 10 min at RT. 10–15 μL of Vectashield + DAPI (Vector Laboratories) was added on top of fixed cells, which were then sealed under a cover slip with nail polish.

#### RNA interference

Cells were diluted to 1.5 × 10^6^ cells/mL in serum-free media. 20 mg of dsRNA generated using the T7 Transcription Kit (ThermoFisher AM1334) was incubated with cells for 45 min. A list of primers used to generate dsRNA can be found in Data S1, Table S3. After 45 min, serum-containing media was added to the RNAi-treated cells. Cells were then incubated for 5 days at 25°C. To confirm the depletion, 1 million cells were harvested and lysed in CSK buffer (10 mM PIPES pH 7.0, 300 mM sucrose, 100 mM NaCl, 3 mM MgCl_2_ with with 2X cOmplete Protease Inhibitor Cocktail EDTA-free (Roche)) for 8 min 2X Laemmli sample buffer (BioRad) was added to lysates and samples were incubated at 95°C for 5 min. Depletions were confirmed by SDS-PAGE followed by western blotting for Elys, ORC2, and Histone H3 as previously described (see western blotting).

### CUT&RUN

CUT&RUN was performed using previously published methods (Ahmad and Spens, 2019; Skene and Henikoff, 2017; Skene et al., 2018). Briefly, 1 million S2 cells were harvested and spun down at 600 × g. Cells were washed with PBS and followed by wash buffer (20 mM HEPES pH 7.5, 150 mM NaCl, 0.1% BSA, with 2X cOmplete^™^ Protease Inhibitor Cocktail EDTA-free (Roche) and 0.6 mM Spermidine). Cells were attached to ConA beads in binding buffer (20 mM HEPES pH 7.9, 10 mM KCl, 1 mM CaCl2, 1 mM MnCl2) for 10 min. Cells were blocked and permeabilized in DBE buffer (wash buffer with 2 mM EDTA and 0.05% digitonin) for 10 min. Cells were then incubated with 1 mg of mab414 antibody (BioLegend) in DBE buffer at 4°C overnight.

After primary antibody incubation, cells were washed twice in DBE buffer. pA-MNase (gift from Kami Ahmad) was diluted 1:400 in DBE buffer and added to cells. pA-MNase was allowed to bind for one hour at room temperature. Cells were then washed twice with wash buffer and suspended in cleavage buffer (wash buffer with 2 mM CaCl_2_). DNA cleavage was carried out for 30 min on ice, then immediately stopped with stop buffer (170 mM NaCl, 20 mM EDTA, 4 mM EGTA). Supernatant containing the cleaved DNA was collected from the cells and treated with RNAse A and Proteinase K. SPRIselect beads (Beckman Coulter) were used to purify the fragmented DNA. To prepare this DNA for sequencing, the NEBNext Ultra II DNA Prep Kit for Illumina (New England Biolabs) was used using according to the manufacturer guidelines and then sequenced using an Illumina NovaSeq6000 for 150 bp PE reads.

#### ChIP-seq

ORC2 ChIP-seq was performed as previously described (MacAlpine et al., 2010). Briefly, 20 million S2 cells for each depletion were harvested and centrifuged at 600 rcf for 5 min. Cells were washed twice with PBS and fixed for 10 min with 1% PFA at room temperature. Crosslinking was quenched by adding glycine to a final concentration of 125 mM and incubating at room temperature for 5 min. Cells were spun down and resuspended in RIPA buffer (50 mM Tris-HCl, 140 mM NaCl, 1 mM EDTA, 1% NP-40, 0.1% Na-Deoxycholate, 0.1% SDS with 2X cOmplete^™^ Protease Inhibitor Cocktail EDTA-free). Cells were incubated for 1 h at 4°C and sonicated using a Diagenode Bioruptor for 4 rounds of 10 cycles (each cycle was 30 s on, 30 s off at max power). After sonication, chromatin extract was cleared by centrifuging at 21,000 rcf for 5 min. The remaining supernatant was used as input for the chromatin immunoprecipitation.

After preparing the chromatin extract, 1 mg of anti-ORC2 antibody was added and allowed to incubate for 2 h at 4°C. Protein A beads were washed with RIPA buffer, added to the extract and incubated for one hour at 4°C. Beads were then washed twice with RIPA buffer, twice with high-salt RIPA buffer (500 mM NaCl), once more with RIPA buffer and once with TE Buffer. To elute protein, beads were incubated with elution buffer (50 mM Tris-HCl pH 8.0, 10 mM EDTA, 1% SDS) at 65°C for 15 min. Protein-DNA cross links were reversed by incubating at 65°C overnight. To recover DNA, samples were RNase A and Proteinase K treated, and phenol:-chloroform extracted. Next, the DNA was isopropanol precipitated. Once the DNA was purified, the NEBNext Ultra II DNA Prep Kit for Illumina (New England Biolabs) was used to prepare the samples for next-generation sequencing. Barcoded libraries were sequenced using an Illumina NovaSeq for 150 bp PE reads.

#### ATAC-seq

For each depletion, 50,000 cells were harvested and washed with PBS. ATAC-seq was performed as described previously (Buenrostro et al., 2015) using an ATAC-seq kit (Active Motif) as described by the manufacturer. Briefly, cells were resuspended in cold lysis buffer and centrifuged at 1000xg for 10 min at 4°C. The cell pellet was resuspended in tagmentation buffer and the reaction was incubated at 37°C for 30 min. Tagmented DNA was purified and used to generate sequencing libraries following manufacturer’s protocol. Libraries were sequenced with an Illumina NovaSeq6000.

#### Flow cytometry

To generate cell cycle profiles for RNAi-treated cells, 10 million cells were first pulsed with 20 mM EdU for 20 min after five days of RNAi treatment. Next, cells were washed twice with PBS and fixed overnight in ice-cold 70% ethanol. After fixation, cells were again washed with PBS and permeabilized for one hour at room temperature with PBX (PBS with 0.1% Triton X-100). Incorporated EdU was click-labeled with an Alexa Fluor 555 Azide (Invitrogen) by incubating with 4 mM CuSO_4_ and 2 mg/mL sodium ascorbate in PBS for 30 min at room temperature. Once clicked labeled, cells were washed twice with PBX and DAPI stained overnight. For the cell cycle analysis in Figure S4B, three biological replicates were performed and the percent of cells in each phase of the cell cycle was quantified.

To quantify the amount of chromatin bound ORC and Histone H2B in nuclei, 50 million cells were harvested after each depletion. The protocol was adapted from Matson et al. (2017). Cells were thoroughly washed with PBS and then lysed in cold CSK buffer supplemented with 0.5% Triton X-100 and 2X cOmplete^™^ EDTA-free Protease Inhibitor Cocktail for eight minutes on ice. PBS with 1% BSA was added to lysates and nuclei were pelleted by centrifugation at 2000xg for three minutes. Nuclei were then fixed with 4% PFA in PBS for 15 min at room temperature. After fixation, PBS with 1% BSA was added and fixed nuclei were pelleted by centrifugation at 2000xg for 7 min. Nuclei were washed once with PBS supplemented with 1% BSA and 0.1% NP40 (Blocking Buffer). Nuclei were incubated overnight at 4°C with either anti-ORC2 antibody or anti-Histone H2B (Abcam cat #52484) diluted 1:200 in blocking buffer. After the primary antibody incubation, nuclei were washed with blocking buffer and then incubated with anti-rabbit antibody conjugated to Alexa Fluorophore 568 (ThermoFisher) diluted 1:500 for two hours at room temperature. Nuclei were then washed twice with blocking buffer and DAPI stained overnight.

DNA content, EdU intensity and ORC2 intensity were determined using a BD LSRII flow cytometer. Flow cytometry data was analyzed and plotted using FlowJo (BD Biosciences). For an example of gating for these experiments, see Figure S3C. For quantifying the ORC2 intensity per nuclei for, 500 nuclei from three replicates were randomly selected and pooled for a total of 1500 nuclei for each depletion. To determine statistical significance, a one-way ANOVA was performed with a post-hoc Dunnett’s test comparing each depletion to the negative control (GFP).

#### Immunofluorescence

Cells were first treated with RNAi for 4 days. After four days, 1–3 million cells were then treated with for 24 h with 1.2 μM aphidicolin in PBS (Millipore Sigma cat#: A0781). This was done to be consistent with our previous depletions by still ensuring a 5-day RNAi treatment. Cells were attached to Concanavalin A coated slides for one hour at room temperature. Cells were washed with PBS and then fixed with 4% PFA for 15 min and permeabilized with permeabilization solution (0.5% Triton X-100) for 8 min. After briefly rinsing in PBS, cells were blocked for 30 min in TBS with 0.1% Tween 20 (TBST) supplemented with 2% Normal Goat Serum (Sigma Aldrich). Histone H2AvD phosphoS137 antibody (Rockland cat #: 600-401-914) was diluted 1:50 in TBST and incubated overnight at 4°C. Next, cells were washed three times with TBST for 5 min each and incubated with Alexa fluorophore 568-conjugated anti-rabbit secondary (ThermoFisher cat#: A-11011), diluted 1:200 for one hour at room temperature. Cells were washed thrice in TBST, DAPI stained and mounted with Vectashield. To determine the cell cycle impact of aphidicolin treatment, cells were RNAi treated for 4 days, and then treated with 1.2 μm aphidicolin for one day for a total of a 5 days of depletion. On the fifth day, cells were pulsed with 20 μM EdU for 20 min, and cells were fixed and click-labeled as previously described (see flow cytometry). The percent of cells in each stage of the cell cycle was quantified for two biological replicates.

For each biological replicate, slides for each depletion were imaged at 40X with the same intensity and exposure time for each channel. To quantify the ɣH2Av signal, Nikon’s NIS Elements software was used to generate regions of interests (ROIs) using DAPI to define the ROI. Mean TxRed (ɣH2Av) and DAPI intensity for each ROI was determined for 300 cells per replicate (600 cells total). To account for differences in DNA content, ɣH2Av intensity was normalized to DAPI intensity. A one-way ANOVA with a post-hoc Dunnett’s test was performed for either the untreated group or the treated group (1.2μM aphidicolin). To determine the effect of treatment within each depletion, a parametric T-test was performed.

To quantify the effects of each depletion on mitosis, immunofluorescence using an anti-phospho-histone H3 (Ser10) antibody (Sigma cat #: 06–570) was performed. Cells were RNAi-treated as previously described (see RNA interference), permeabilized for one hour with PBS supplemented with 0.1% Triton X-100 and fixed with 4% PFA for 15 min. After fixation, cells were incubated with primary antibody diluted 1:200 overnight. Following incubation, cells were washed three times with PBS and then incubated with Alexa fluorophore 568-conjugated anti-rabbit secondary (ThermoFisher cat#: A-11011), diluted 1:200 for two hours at room temperature. Cells were again washed three times with PBS, DAPI stained, and mounted with Vectashield. Two biological replicates were performed. For each replicate, 400 cells were imaged using previously described methods and the percent of cells positive for phosphor-histone H3 staining was determined.

### QUANTIFICATION AND STATISTICAL ANALYSIS

#### Random permutation analysis

Peaks were downloaded for histone modification and transcription factor binding sites identified by ChIP-chip or ChIP-seq in Drosophila from modENCODE (Celniker et al., 2009; Contrino et al., 2012). All available ChIP-seq data in S2 cells were considered in addition to previously published ORC2 (Eaton et al., 2011) and nucleoporin peaks (Gozalo et al., 2020; Pascual-Garcia et al., 2017). For each ChIP-seq factor, the amount of base-pair overlap was calculated between the given factor and ORC2 peaks. A permutation-based technique was used to determine whether the observed amount of overlap was more or less than expected by chance. Briefly, an empirical p value was calculated for the observed amount of overlap by comparing to a null distribution obtained by randomly shuffling regions throughout the genome and calculating the amount of overlap in each permutation. The p values were adjusted for multiple testing using the Bonferroni correction. In this analysis, the location of the ORC2 peaks was maintained and the locations of the histone modification or transcription factor binding peaks were shuffled. The length distribution of the shuffled peaks was matched to the original set and excluded all gap and ENCODE blacklisted regions from consideration. 1000 permutations were performed for each marker and ORC2 pair. To determine factors that were specific for ORC2 or Elys, the same analysis was performed for either Elys binding sites with either Elys alone or binding sites that contained both Elys and ORC2 peaks. The difference in Log2 Fold Enrichment was also quantified in Figure S3.

#### Sequencing analysis

Previously published data generated by ChIP-seq in Drosophila S2 cells was retrieved for ORC2 (Eaton et al., 2011). Elys, Nup107, Nup93, (Gozalo et al., 2020), Nup98 (Pascual-Garcia et al., 2017), mab414 data was generated by CUT&RUN (see CUT&RUN methods). Sequencing reads were aligned to dm6 with Bowtie2 (Langmead and Salzberg, 2012) using the pre-set –very sensitive-local. Duplicate reads were flagged after alignment with Picard: MarkDuplicates (Broad Institute) using Galaxy (Afgan et al., 2016). Coverage files were generated using Deeptools: BamCoverage (Ramírez et al., 2016) with the following options: 1X normalization, bin size = 50 bps, effective genome size = dm6. Genomic coverage was visualized using the UCSC Genome Browser (Kent et al., 2002) as shown in Figure 2A. For peak comparisons, previously published peak files were used (Eaton et al., 2011; Gozalo et al., 2020; Pascual-Garcia et al., 2017). For mab414, statistically significant peaks over an IgG negative control were called using MACS2 (Feng et al., 2012). Deeptools: plotHeatmap was used to generate the mean ChIP-seq signal plots and heatmaps centered on ORC2 peaks as shown in Figure 2B.

The ATAC-Seq and ORC2 ChIP-seq data in Figure 3 was processed similar as above with minor differences. To generate the coverage plots for visualization, the ATAC-Seq data was normalized by CPM (counts per million) with a bin size = 50. To scale the ORC2 ChIP-seq data to the background signal, 25,000 genomic regions, each 250 base pairs long, were randomly selected. The total reads within the randomly selected regions for each depletion was determined and scaled down to the depletion with the fewest reads. The scaled coverage files were plotted in the UCSC Genome Browser for both Figures 3C and 3E. For both ORC2 ChIP-seq and ATAC-seq, the mean signal was determined using Deeptools: plotProfile for each set of peaks. To generate shuffled ORC2 peaks, ORC2 peaks were randomly distributed across the genome, and the number of peaks and the length of each peak were kept the same using BedTools: ShuffleBed.

For the plots generated in Figure 3D, both ChIP-seq replicates were first scaled to background as above. Replicates were then scaled again for visualization purposes by determining the maximum signal in the GFP depletion and then scaling all the data for all depletions by the same scaling factor. This was performed to account for the different number of reads and differences in signal intensity between the two replicate experiments.

#### Statistics

For all statistics, relevant p values are denoted within the respective figure legends. Error bars in all bar graphs show the standard deviation. For the volcano plot in Figure 1B, Fold enrichment was calculated by dividing spectrum counts for GFP IP by the negative control. P values were calculated by performing a Fisher’s Test for each individual protein. p-values less than 0.00010 were rounded for simplicity. For the statistical test in Figures 2E and 3B, One-Way ANOVA with a post-hoc Dunnett’s test was used to determine statistical significance relative to the respective negative control. For Figure 4B, black bars indicate a One-Way ANOVA with a post-hoc Dunnett’s test comparing each depletion to negative control (GFP). Pink bars indicate a parametric T-test performed between each depletion comparing the untreated cells to the aphidicolin treated cells. 300 cells from two biological replicates were randomly selected for the quantification.

## Supplementary Material

MMC3

MMC2

Data S1

## Figures and Tables

**Table:** KEY RESOURCES TABLE

REAGENT or RESOURCE	SOURCE	IDENTIFIER
Antibodies		
Rabbit Anti-ORC2 Antibody	This Paper	N/A
Rabbit Anti-Elys Antibody	This Paper	N/A
HRP Rabbit Anti-Histone H3 Antibody	Abcam	Cat#: ab21054; RRID:AB_880437
Rabbit Anti-phospho-Histone H3 (Ser10) Antibody	Sigma-Aldrich	Cat#: 06-570; RRID:AB_310177
Rabbit Anti-Histone H2AvD phospho137 Antibody	Rockland	Cat#: 600-401-914; RRID:AB_828383
Mouse Anti-Nuclear Pore Complex Proteins Antibody (mab414)	BioLegend	Cat#: 902901; RRID:AB_2565026
Mouse Anti-H2B Antibody	Abcam	Cat#: ab52484; RRID:AB_1139809
Rabbit IgG	Sigma-Aldrich	Cat#: I5006; RRID:AB_1163659
Alexa Fluor 568 Goat Anti-Rabbit Antibody	ThermoFisher	Cat#: A11011; RRID:AB_143157
Alexa Fluor 488 Goat Anti-Mouse Antibody	ThermoFisher	Cat#: A11029; RRID:AB_2534088
Peroxidase-AffiniPure Donkey Anti-Rabbit	Jackson ImmunoResearch	Cat#: 712-035-153; RRID:AB_2340639
Bacterial and virus strains		
Rosetta ^™^ 2 (DE3) Competent Cells	Novagen	Cat#: 71400
Chemicals, peptides, and recombinant proteins		
cOmplete^™^ Protease Inhibitor Cocktail EDTA-free	Roche	Cat#: 04693159001
Benzonase ^®^ Nuclease	Fisher Scientific	Cat#: 70-664-3
GFP Trap ^®^ Magnetic Agarose	Chromotek	Cat#: gtma-20
SPRIselect Beads	Beckman Coulter	Cat#: B23317
2× Laemmli Sample Buffer	Bio-Rad	Cat#: 1610737
Alexa Fluor 555 Azide	Invitrogen	Cat#: A20012
RNAse A	Sigma-Aldrich	Cat#: R4875
Proteinase K	Sigma-Aldrich	Cat#: P4850
Aphidicolin	Sigma-Aldrich	Cat#: A0781
Normal Goat Serum	Sigma-Aldrich	Cat#: G9023
Vectashield+DAPI	Vector Laboratories	Cat#: H-1000
Maxima H Minus Reverse Transcriptase	ThermoFisher Scientific	Cat#: EP0752
Critical commercial assays		
MagExtractor PCR & Gel Clean Up Kit	Toyobo	Cat#: NPK-601
MEGAscript ^™^ T7 Transcription Kit	ThermoFisher Scientific	Cat#: AM1334
4–15% Mini-PROTEAN^™^ TGX Stain-Free^™^ Protein Gels	Bio-Rad	Cat#: 4568086
DNA Clean & Concentrator-100 kit	Zymo Research	Cat# D4029
NEBNext Ultra II DNA Prep Kit for Illumina	New England BioLabs	Cat#: E7370
ATAC-Seq Kit	Active Motif	Cat#: 53150
Deposited data		
modEncode ChIP-ChIP or ChIP-seq S2 cell data	Celniker et al. (2009); Contrino et al. (2012)	N/A
ORC2 ChIP-seq peaks in S2 cells	Eaton et al. (2011)	GSE20887
Nucleoporin peaks in S2 cells	Gozalo et al. (2020); Pascual-Garcia et al. (2017)	GSE136117; GSE94922
Raw and processed sequencing data	This study	GSE199896
Processed mass spectrometry data	This study	ProteomeXchange: PXD033045
Raw western blot images	This study	Mendeley Data: https://doi.org/10.17632/z4f2tkn4gs.1
Experimental models: Cell lines		
Drosophila S2 cells	Drosophila Genomics Resource Center	Cat#: 181
Experimental models: Organisms/strains		
WT: *Oregon R* flies	N/A	N/A
ORC2-GFP flies	Gift from Shelby Blythe	endogenously tagged *ORC2*
Oligonucleotides		
Oligopaint Primers (See Data S1, Table S2)	This Study	N/A
OligoPaint PCR Amplification Forward Primer: 5′ GCGTTAGGGTGCTTACGTC-3′	This Study	N/A
OligoPaint PCR Amplification Reverse Primer: 5′ CACCTCCGTCTCTCACCT-3′	This Study	N/A
Oligopaint Fluorescent Secondary Probe covalently lined to Alexa Fluor 488: AAGCACCCTAACGCTTCACGATCCAT	This Study	N/A
Primers used to generate dsRNA for RNA interference (See Data S1, Table S3)	This Study	N/A
Recombinant DNA		
Plasmid: pLM302 His-MBP-ORC2	This study	His-MBP-ORC2 fusion under T7 promoter
Plasmid: pLM302 His-MBP-Elys	This study	His-MBP-Elys fusion under T7 promoter
Software and algorithms		
GraphPad Prism	Software	https://www.graphpad.com/scientific-software/prism/
FlowJo	Software	https://www.flowjo.com/
RStudio	Software	https://www.rstudio.com/
Nikon Elements	Software	https://www.microscope.healthcare.nikon.com/products/software/nis-elements
Bowtie2	Software	http://bowtie-bio.sourceforge.net/bowtie2/index.shtml
Picard	Software	https://broadinstitute.github.io/picard/
Deeptools	Software	https://deeptools.readthedocs.io/en/develop/
MACS2	Software	https://pypi.org/project/MACS2/
Bedtools	Software	https://bedtools.readthedocs.io/en/latest/index.html
UCSC Genome Browser	Software	https://genome.ucsc.edu/
Galaxy	Software	https://usegalaxy.org/

## Highlights

- ORC2 interacts with the Nup107-160 subcomplex of the nuclear pore
- ORC2 and subunits of the Nup107-160 subcomplex bind the same genomic regions
- Depletion of the nucleoporin Elys reduces ORC2 loading onto chromatin
- Depleting Elys sensitizes cells to replication fork stalling
