# Supplementary material for: Nucleoporins facilitate ORC loading onto chromatin
Source: Cell Rep. Author manuscript; Available in PMC 2023 Mar 26. (PMC10040217; doi:10.1016/j.celrep.2022.111590)
Supplement: Data S1 [file NIHMS1848904-supplement-Data_S1.pdf]

Cell Reports, Volume *41*

Supplemental information

Nucleoporins facilitate ORC loading onto chromatin

Logan Richards, Christopher L. Lord, Mary Lauren Benton, John A. Capra, and Jared T. Nordman

A

Group	Protein	ORC2-GFP Rep. #1	ORC2-GFP Rep. #2	ORC2-GFP Rep. #3	negative control Rep. #1	negative control Rep. #2	negative control Rep. #3	Comparison of abundance* (p-value)
ORC	**ORC2**	316	339	323	4	0	2	< 0.00010
ORC	ORC1	109	80	123	2	0	0	< 0.00010
ORC	ORC3	463	446	452	0	0	0	< 0.00010
ORC	ORC4	58	43	42	0	0	0	< 0.00010
ORC	ORC5	113	72	69	0	0	0	< 0.00010
ORC	ORC6	148	151	155	0	0	0	< 0.00010
Nup107-160 Subcomplex	Elys	16	31	96	6	2	2	< 0.00010
Nup107-160 Subcomplex	Nup96-98	5	6	9	0	0	0	< 0.00010
Nup107-160 Subcomplex	Nup75	4	6	10	1	0	1	0.00067
Nup107-160 Subcomplex	Nup160	4	5	7	0	1	0	0.0010
Nup107-160 Subcomplex	Nup133	3	4	7	0	0	1	0.0028
Nup107-160 Subcomplex	Nup107	6	4	6	1	1	1	0.013
Nup107-160 Subcomplex	Nup44A	20	12	7	27	7	9	0.051
Nup107-160 Subcomplex	Sec13	7	4	5	8	5	4	0.20
Nup107-160 Subcomplex	Nup43	0	0	2	0	0	0	0.33
Nup107-160 Subcomplex	Nup37	0	0	0	0	0	0	n.a.
Othr Nucleoporins	Nup205	9	3	7	0	0	0	0.0036
Othr Nucleoporins	Nup93	1	1	3	0	0	0	0.061
Othr Nucleoporins	Nup358	6	11	16	4	8	8	0.27
Othr Nucleoporins	Nup154	3	3	2	3	0	6	0.27
Othr Nucleoporins	Nup88	2	1	2	0	0	3	0.53

*Fisher's Exact Test

B

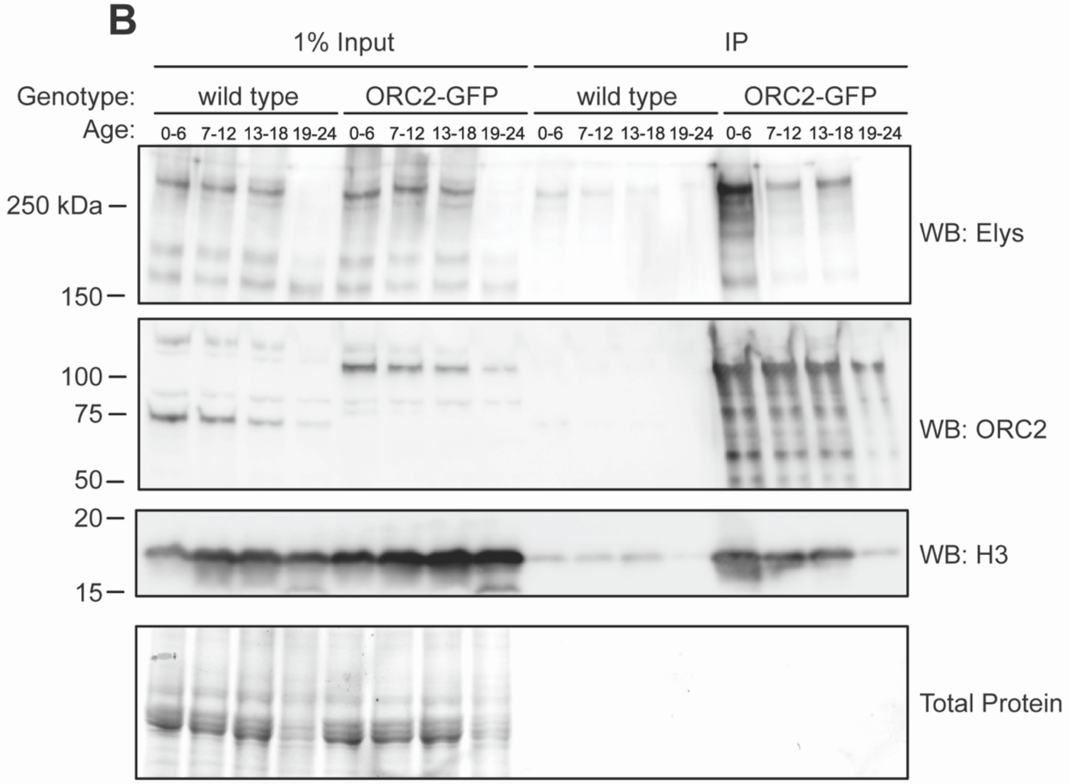

Supplemental Figure 1. ORC2-GFP immunoprecipitation enriches for components of the Nup107-160 subcomplex of the nuclear pore. (A) Table with peptide counts for three biological replicates of anti-GFP IP mass spectrometry done in either ORC2-GFP or negative control embryos as in Figure 1. Embryos were aged 16-24 hours. P-value was calculated by performing a Fisher's Test. (B) Western blot of anti-GFP IP done in ORC2-GFP embryos throughout embryonic development. Western blots done using anti-Elys, anti-ORC2, or anti-Histone H3 antibodies. IPs were performed on embryos from indicated ages after egg laying (in hours). Total protein loaded for each sample shown in bottom box.

A

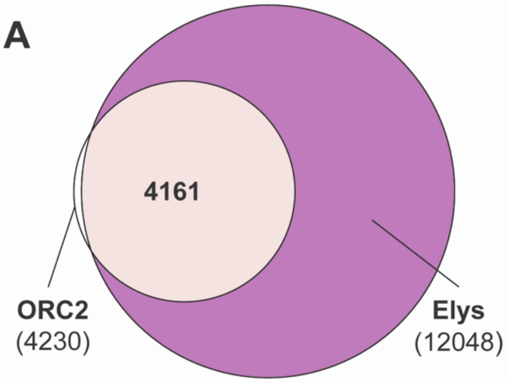

B

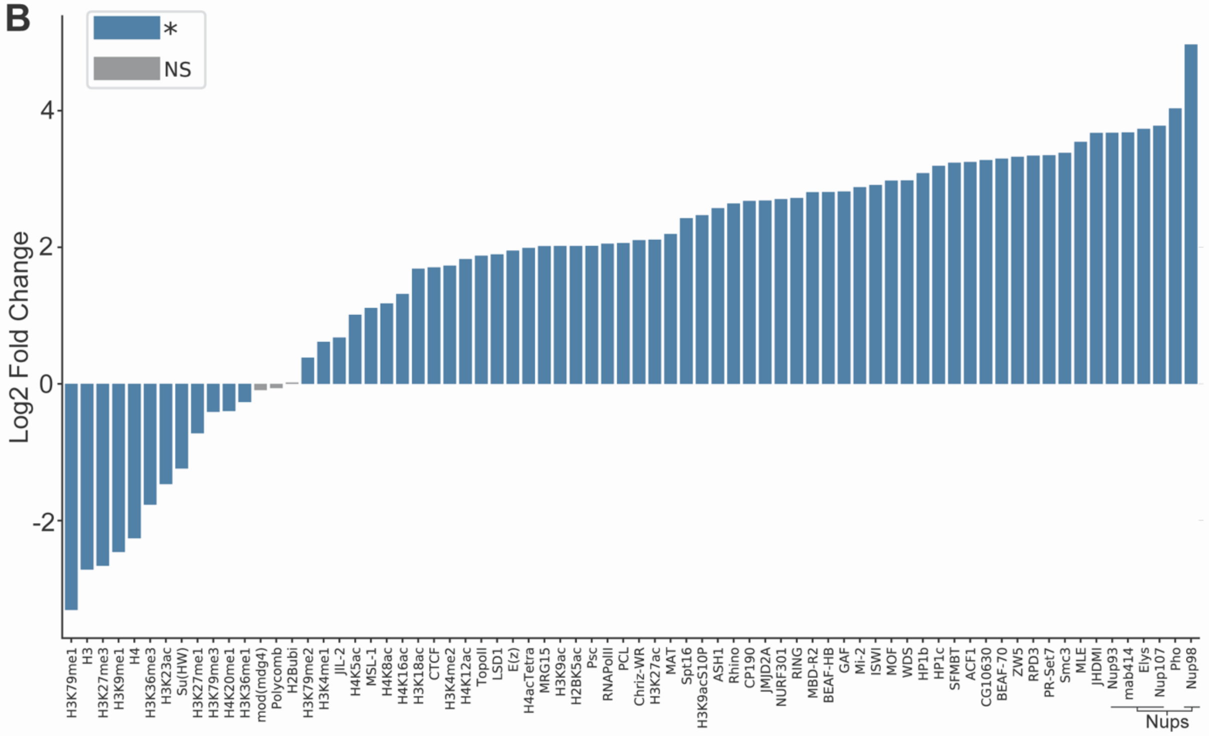

C

Site Name	Coordinates
Pos. Control	chr2L:1352742-1360992
Neg. Control	chr3R:19335000-19345000
ORC2 Site 1	chr3R:26879000-26888250
ORC2 Site 2	chr3L:14405500-14415500
ORC2 Site 3	chr3R:13644500-13654500
ORC2 Site 4	chr2L:12000000-12008000
ORC2 Site 5	chr2R:11600000-11611000
ORC2 Site 6	chrX:12727000-12742000
ORC2 Site 7	chrX:13822000-13830500

D

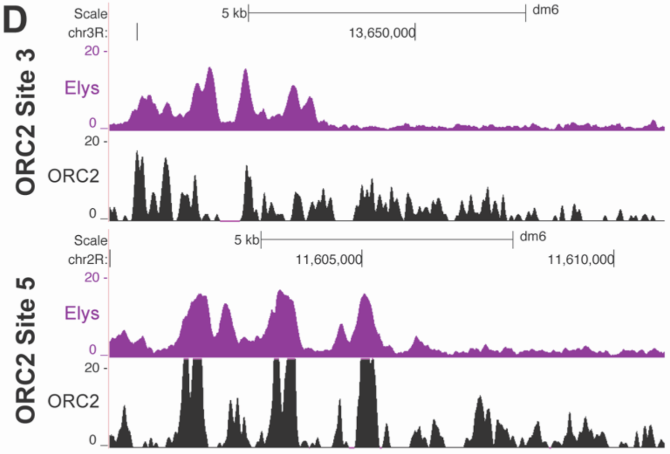

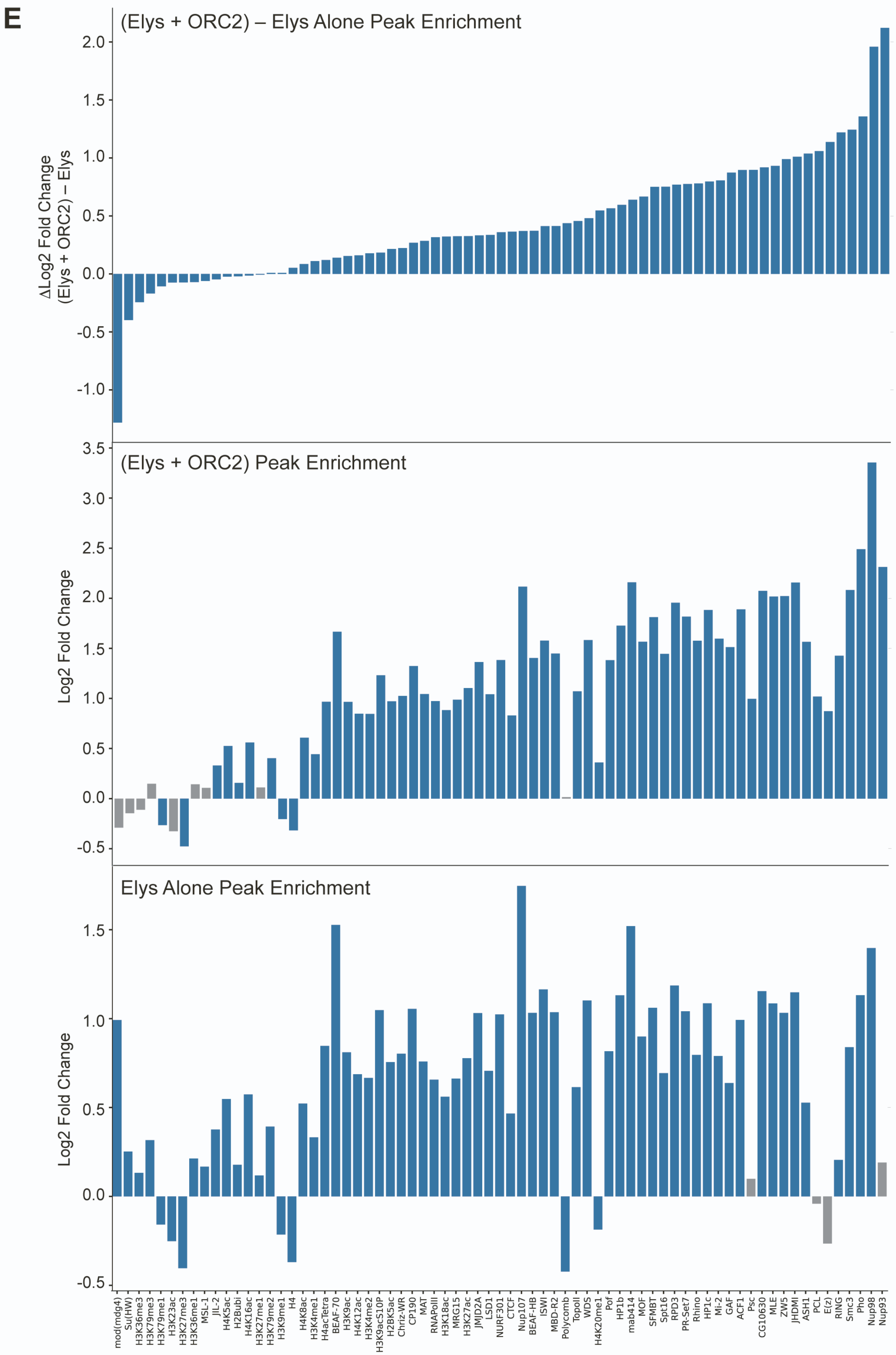
E
(Elys + ORC2) – Elys Alone Peak Enrichment
ΔLog2 Fold Change (Elys + ORC2) – Elys
2.0
1.5
1.0
0.5
0.0
-0.5
-1.0
(Elys + ORC2) Peak Enrichment
Log2 Fold Change
3.5
3.0
2.5
2.0
1.5
1.0
0.5
0.0
-0.5
Elys Alone Peak Enrichment
Log2 Fold Change
1.5
1.0
0.5
0.0
-0.5
mod(mdg4)
Su(HW)
H3K36me3
H3K79me3
H3K79me1
H3K23ac
H3K27me3
H3K36me1
MSL-1
JIL-2
H4K5ac
H2Bubi
H4K16ac
H3K27me1
H3K79me2
H3K9me1
H4
H4K8ac
H3K4me1
H4acTetra
BEAF-70
H3K9ac
H4K12ac
H3K4me2
H3K9acS10P
H2BK5ac
Chriz-WR
CP190
MAT
RNAPolII
H3K18ac
MRG15
H3K27ac
JMJD2A
LSD1
NURF301
CTCF
Nup107
BEAF-HB
ISWI
MBD-R2
Polycomb
TopoII
WDS
H4K20me1
Pof
HP1b
mab414
MOF
SFMBT
Spt16
RPD3
PR-Set7
Rhino
HP1c
Mi-2
GAF
ACF1
Psc
CG10630
MLE
ZW5
JHDMI
ASH1
PCL
E(z)
RING
Smc3
Pho
Nup98
Nup93

Supplemental Figure 2. Nups are enriched at ORC2 binding sites. (A) Venn diagram visualizing peak overlap between ORC2 (white) and Elys (purple). Number in parenthesis is the total number of peaks. Bold number is the number of ORC2 peaks that overlap with Elys peaks (4161 out of 4230). (B) Bar graph visualization depicting the log2 fold enrichments for data in Fig. 2C. Blue bars denote chromatin marks, transcription factors or nucleoporins that had a statistically significance correlation (positive or negative) with ORC2 peaks. Gray bars denote those that had a nonsignificant correlation. (C) Table containing site names and genomic locations of oligopaint probes for Fig. 2. (D) Representative genome browser view for one biological replicate of Elys and ORC2 binding sties used for probes in oligopainting visualized in Fig. 2D (ORC2 sites 3 and 5). (E) Top: Bar graphs indicate difference in log2 fold enrichment (ΔLog2 Fold Change) between peaks containing both Elys and ORC2 (Elys+ORC2) and Elys only peaks for chromatin-associated factors from previously published data. Middle: Bar graph visualization depicting log2-fold enrichment for same set of factors for peaks containing both Elys and ORC2. Bottom: same as middle panel with peaks enriched for Elys but not ORC2. Blue bars: statistically enriched. Gray bars: not significant.

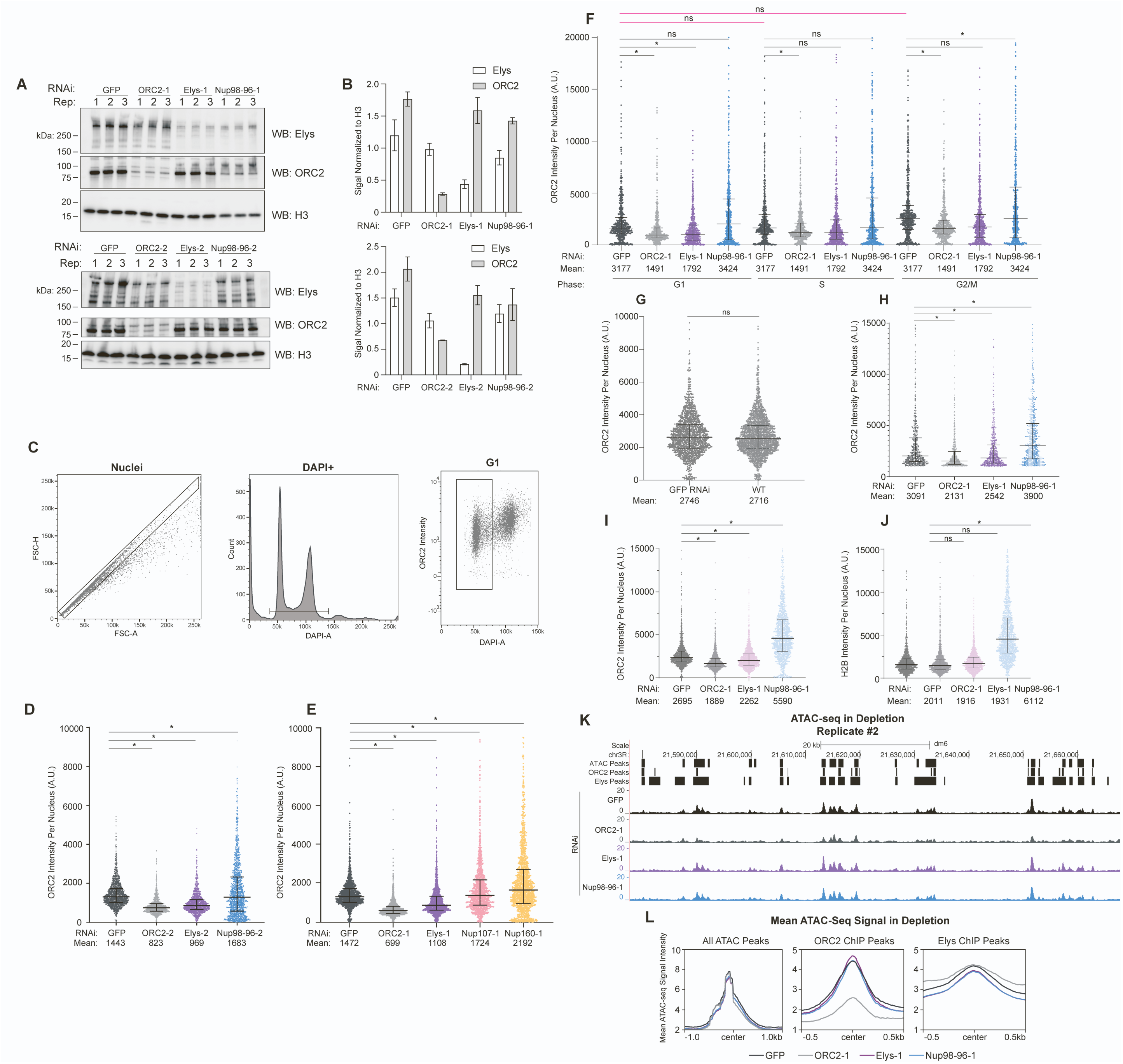

Supplemental Figure 3. Not all nucleoporins contribute to ORC chromatin association. (A) Western blots using anti-Elys, anti-ORC2 or anti-Histone H3 antibodies on samples prepared from cells treated with the indicated RNAi. Shown are three biological replicates. (B) Quantification of (A). Western blot signal for Elys (white) or ORC2 (gray) normalized to H3 for each depletion. (C) Gating example for G1 nuclei. Single nuclei isolated in the first gate indicated by the black box. DAPI positive nuclei were selected, indicated by black line, to generate a horseshoe plot. The first DAPI peak, shown as a black box in the third panel, was used for quantification. (D) Quantification of ORC2 intensity per nucleus using a second set of dsRNAs against ORC2,

Elys, and Nup98-96. Each depletion shown contains 1500 nuclei taken from one biological replicate. Asterisk denotes a P value of < 0.0001 and was determined by a one-way ANOVA with a post-hoc Dunnett's test. (E) Same as (D) but with RNAi done using dsRNAs: GFP, ORC2-1, Elys-1, Nup107-1, and Nup160-1. Each depletion shown contains 750 nuclei randomly selected and pooled from two biological replicates for 1500 nuclei total. (F) ORC2 intensity quantified for each cell cycle phase. 600 nuclei from three biological replicates were pooled for the analysis. Asterisk denotes P-value of < 0.05 determined by a one-way ANOVA with a post-hoc Dunnett's test. NS: no significance. Black bars indicate comparison between GFP vs. depletion for each cell cycle phase. Pink bars indicate comparison of GFP vs. GFP for each cell cycle. (G) Chromatin bound ORC2 levels in GFP RNAi-treated G1 nuclei compared to untreated wild type G1 nuclei. The ORC2 intensity was quantified in 1500 G1 nuclei taken from two biological replicates. NS: no significance determined by an unpaired parametric T-test. (H) ORC2 chromatin association is reduced in Elys-depleted nuclei positive for ORC2 signal. Only nuclei with an ORC2 intensity greater than 10^3 were used to quantify the ORC2 intensity in G1 nuclei. 600 nuclei from three biological replicates for each depletion were pooled for the analysis. Asterisk denotes P-value of < 0.0001 and was determined by a one-way ANOVA with a post-hoc Dunnett's test. (I) 1500 randomly selected G1 nuclei across two biological replicates were used to quantify the ORC2 intensity per nucleus for each depletion. Asterisk indicates p<0.0001 relative to the negative control by One-Way ANOVA with a post-hoc Dunnett's test. (J) Same as (I) for Histone H2B intensity. Increases in H2B levels correlate with increased ploidy upon Nup98-96 depletion. (K) Representative UCSC genome browser view of ATAC-seq profiles for each depletion. ORC2 binding sites (ORC2 Peaks - defined by Eaton et al., 2011) are indicated by black bars. (L). Quantification of mean ATAC-seq signal for either all ATAC-seq peaks (n=12771), ORC2 ChIP-seq peaks (n=4280) or Elys ChIP-seq peaks (n=12048) (defined by Pascual-Garcia et al., 2017) centered on their respective peaks. Note the scales are different for all ATAC-seq peaks plots. These data are a second biological replicate of ATAC-seq data provided in Figure 3.

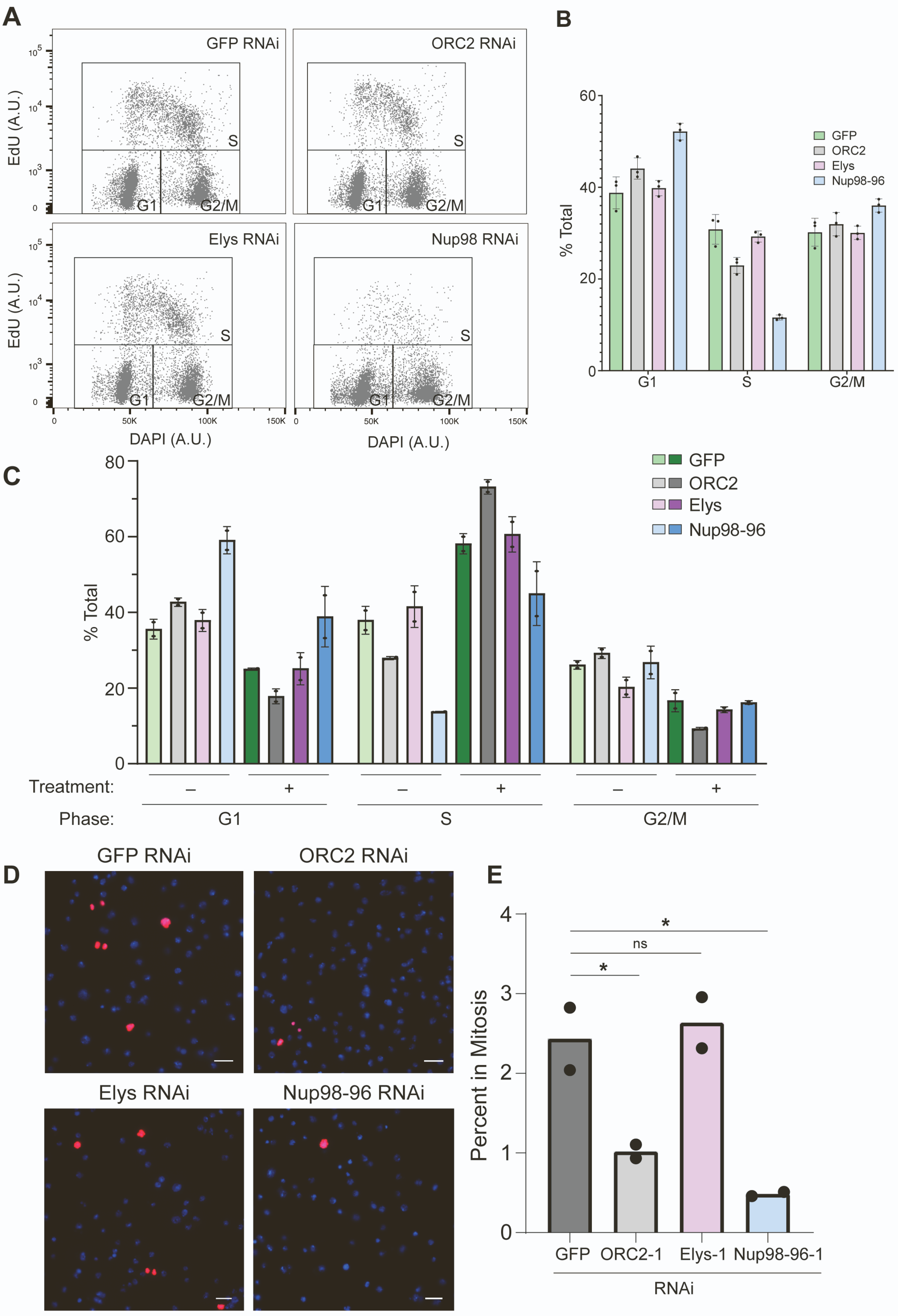
A
GFP RNAi
ORC2 RNAi
Elys RNAi
Nup98 RNAi
EdU (A.U.)
DAPI (A.U.)
S
G1
G2/M
B
% Total
GFP
ORC2
Elys
Nup98-96
G1
S
G2/M
C
% Total
GFP
ORC2
Elys
Nup98-96
Treatment:
–
+
Phase:
G1
S
G2/M
D
GFP RNAi
ORC2 RNAi
Elys RNAi
Nup98-96 RNAi
E
Percent in Mitosis
*
ns
*
GFP
ORC2-1
Elys-1
Nup98-96-1
RNAi

Supplemental Figure 4. Nup depletions differentially affect cell cycle progression (A) Horseshoe plot of RNAi-treated cells. Cells were DAPI stained and EdU pulsed to determine cell cycle phase. Black boxes indicate gating used to quantify percent of cell population within each cell cycle phase. (B) Percentage of cells in each indicated phase of the cell cycle (A). Shown are three biological replicates. Error bars represent the standard error of the mean. (C) Quantification of the effects a low dose of aphidicolin has on cell cycle progression for each depletion. Percentage of cells in each indicated cell cycle phase in either untreated cells (–) or cells treated with 1.2 uM aphidicolin. Data are from two biological replicates. Error bars represent the standard error of the mean. (D) Representative images of phospho-histone H3 (PH3) immunofluorescence performed on RNAi-treated cells. Blue: DAPI. Red: PH3. Scale bar: 10 μM. (E) The percent of PH3 positive cells (percent in mitosis) from two biological replicates was quantified. Approximately 400 cells from each biological replicate were used for quantification. Asterisk denotes P-value < 0.05 determined by a one-way ANOVA followed by a post-hoc Dunnett's Test. NS: no significance.
